# Ladder-like Polymer Brushes Containing Conjugated Poly(Propylenedioxythiophene) Chains

**DOI:** 10.3390/ijms23115886

**Published:** 2022-05-24

**Authors:** Gabriela Grześ, Karol Wolski, Tomasz Uchacz, Justyna Bała, Boris Louis, Ivan G. Scheblykin, Szczepan Zapotoczny

**Affiliations:** 1 Faculty of Chemistry, Jagiellonian University, Gronostajowa 2, 30-387 Krakow, Poland; gabriela.grzes@uj.edu.pl (G.G.); uchacz@chemia.uj.edu.pl (T.U.); justyna.bala@doctoral.uj.edu.pl (J.B.); 2 Division of Chemical Physics and NanoLund, Lund University, 22100 Lund, Sweden; boris.louis@kuleuven.be (B.L.); ivan.scheblykin@chemphys.lu.se (I.G.S.); 3 Molecular Imaging and Photonics, Department of Chemistry, KU Leuven, Celestijnenlaan 200F, 3001 Leuven, Belgium

**Keywords:** polymer brushes, conjugated polymers, ProDOT, photoluminescence, photostability

## Abstract

The high stability and conductivity of 3,4-disubstituted polythiophenes such as poly(3,4-ethylenedioxythiophene) (PEDOT) make them attractive candidates for commercial applications. However, next-generation nanoelectronic devices require novel macromolecular strategies for the precise synthesis of advanced polymer structures as well as their arrangement. In this report, we present a synthetic route to make ladder-like polymer brushes with poly(3,4-propylenedioxythiophene) (PProDOT)-conjugated chains. The brushes were prepared via a self-templating surface-initiated technique (ST-SIP) that combines the surface-initiated atom transfer radical polymerization (SI-ATRP) of bifunctional ProDOT-based monomers and subsequent oxidative polymerization of the pendant ProDOT groups in the parent brushes. The brushes prepared in this way were characterized by grazing-angle FTIR, XPS spectroscopy, and AFM. Steady-state and time-resolved photoluminescence measurements were used to extract the information about the structure and effective conjugation length of PProDOT-based chains. Stability tests performed in ambient conditions and under exposure to standardized solar light revealed the remarkable stability of the obtained materials.

## 1. Introduction

Since the discovery of conjugated polymers (CPs) [1], they have been of interest to many scientific groups thanks to their unique photoelectrical properties. They have attracted especially high interest in recent years due to the significant developments in the synthetic approaches and desired applications [2,3,4]. Taking into account the favorable processability of conductive polymers, possibility of tailoring their structures and functions at the molecular scale, low production costs, and reasonable stability, π-conjugated polymers may find applications in next-generation electronic devices [5], such as photovoltaic organic solar cells [6], bioelectronics [7], energy storage devices [8], energy conversion systems [9], and electrochemical transistors [10].

In these applications, the optical properties of the CPs as well as their photostability are crucial. In general, CPs are often prone to photodegradation [11], photobleaching [12], photoblinking [13], inter- or intramolecular charge transfer [14], and photo-oxidation defects [15]. All these phenomena are detrimental to their applications in devices and thus, it is important to characterize and understand them so they can be diminished or even eliminated.

Polythiophenes, one of the most important classes of conjugated polymers, have been extensively investigated for their great optical properties [16,17,18,19]. Indeed, polythiophene and its derivatives are typically characterized by a relatively stable conductivity level and optical transparency, which are relevant for various applications [20]. However, considerable attention has recently been focused on alkylenedioxy-substituted thiophenes, aiming at improved chemical stability. The substituted monomers only have two reactive hydrogens located in the 2,5 positions on opposite sides of the sulfur atom in the conjugated thiophene ring, ensuring that the resulting polymer chains are characterized by a high stiffness and long effective conjugation length. Furthermore, the lack of residual hydrogen atoms attached directly to the conjugated backbone contributes to the improved chemical stability of such substituted thiophenes [21].

Moreover, the 3,4-substitution also lowers the oxidation potential at which thiophene-based monomers undergo polymerization [22]. Among them, the most studied polymer is poly(3,4-ethylenedioxythiophene) (PEDOT) [23,24], which is one of the most chemically stable conjugated polymers, demonstrating high conductivity in the doped state (3000 S/cm or more, which is only slightly lower than the conductivity of metals) [25,26]. PEDOT intermixed and stabilized by poly(styrenesulfonate) (PSS) is probably the most popular and widely used polymer mixture in the field of conducting polymers—PEDOT:PSS [27]. Besides the outstanding properties of PEDOT:PSS, such as its high conductivity and stability, PEDOT:PSS thin films typically show much higher in-plane than out-of-plane conductivity, which is unfavorable for certain applications, e.g., in fabricating sensors, nanoelectronics, molecular rectifiers, ordered photovoltaic systems, etc., [28,29].

Poly(3,4-propylenedioxythiophene) (PProDOT) is structurally similar to PEDOT, only differing in an extra carbon atom in the dialkoxy ring. PProDOT polymers are similarly stable to PEDOT and highly conductive [30]. The two oxygen atoms present in EDOT and ProDOT monomers supply additional electron density to the conjugated molecular backbone, influencing the efficiency of charge transport [21].

The doping process increases the conductivity of the semiconducting polymers, through the removal of electrons (p-type doping) or by introducing additional electrons (n-type doping), enhancing the hole or electron-type conduction mechanism [31]. For p-type doping, some electron acceptors such as I_2_ [32], FeCl_3_ [33], tetrafluorotetracyano-quinodimethane and its derivatives [34,35] are commonly used.

Most applications require the formation of conjugated polymer layers on the surface. Polymer films are usually created using spin-coating [36], drop-casting [37], or spray-coating [36] methods. Nevertheless, such approaches are hardly applicable for native PEDOT or PProDOT, which are generally insoluble and infusible [38]. Hence, the formation of ordered or self-assembled systems based on such insoluble but very stable conductive polymers is highly demanded. Very attractive candidates that may meet these requirements are surface-grafted conjugated polymer brushes (CPBs). CPBs are composed of conjugated polymer chains covalently attached to the substrate [39]. In the case of a high-grafting-density CPBs, the architecture ensures a highly ordered and extended conformation of polymer chains oriented perpendicularly with respect to the substrate [40]. However, high grafting densities can only be achieved by surface-initiated polymerizations that are compatible with the “grafting from” approach [41]. The most commonly used method for the synthesis of CPBs in accordance with the “grafting from” approach is surface-initiated Kumada Catalyst Transfer Polycondensation (SI-KCTP). Although this powerful method was used for the synthesis of certain CPBs, such as poly(3-alkylthiophenes) [42,43] or poly(p-phenylene) [44], it is less useful when it comes to the polymerization of bulky monomers with more complex architectures. Furthermore, to the best of our knowledge, neither PEDOT nor PProDOT brushes were synthesized by SI-KCTP. It is worth emphasizing that CPBs demonstrating high stability and directional conductivity are potentially attractive in applications such as: photovoltaic solar cells, molecular rectifiers, sensors, or single-molecule electronics [2]. CPBs are also useful for the investigation of charge transport occurring on the molecular scale, providing a fundamental understanding of this process [2,39].

Although there have been reports on tethering PEDOT chains to a substrate, mainly to improve the adhesion of the polymer films to inorganic solid surfaces [45,46,47], no ordered grafted polymer brush structures have been shown. Moreover, there was also an attempt to create PEDOT-like brushes via surface-initiated ring-opening metathesis polymerization (SI-ROMP) followed by electropolymerization [48]. Due to the limited data presented in a short communication and the lack of any follow-up publications, it is not convincing that the proposed approach results in the formation of densely grafted brushes. Thus, to the best of our knowledge, there have been no comprehensive reports in the literature on PEDOT or PProDOT surface-grafted polymer brushes obtained by a surface-initiated process.

In this work, we present the synthesis and physicochemical characteristics of PProDOT-based surface-grafted brushes. In order to obtain the brushes, we used the self-templating surface-initiated polymerization (ST-SIP) method, which enables the formation of densely packed and well-ordered brushes with ladder-like architectures [40,49,50,51,52,53]. In this approach, surface-initiated reversible deactivation radical polymerizations (SI-RDRPs) are used at first to prepare the so-called parent brushes (surface-grafted multimonomers) with pendant functional groups that could be converted into conjugated chains by means of template polymerization. In this report, we used surface-initiated atom transfer radical polymerization (SI-ATRP) to obtain the linear brushes with pendant ProDOT groups. In the next step, ProDOT groups were polymerized by template oxidative polymerization in the presence of FeCl_3_, allowing the formation of the conjugated ladder-like polymer brushes. The synthesized conjugated PProDOT-based brushes, which were done so for the first time, exhibited very good stability in their native form and promising optical properties.

## 2. Results and Discussion

### 2.1. Synthesis of ProDOT-MM Monomer

**Figure 1:**
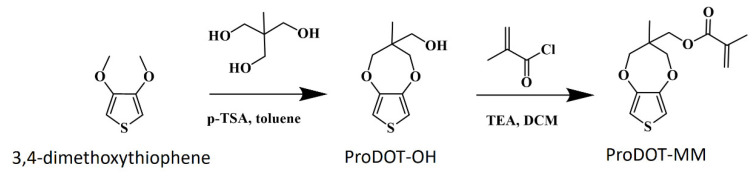
Synthetic route to ProDOT-MM monomer.

The two-step synthesis of the new monomer (3-methyl-2,4-dihydrothieno [3,4-b][1,4]dioxepin-3-yl)methyl 2-methylprop-2-enoate (abbreviated as ProDOT-MM) was carried out according to the following scheme (Figure 1):

The reaction was performed following the modified procedure previously reported by Kim et al. [54]. These two reactions allowed us to obtain a new bifunctional monomer ProDOT-MM with methacryloyl and 3,4-propylenedioxythiophene groups. The final structure was confirmed by NMR, LCMS, and FT-IR spectroscopies (see Section 3 and Supplementary Materials). The choice of such a monomer structure was motivated by the reported higher stability of PEDOT and PProDOT compared to polythiophene-based polymers. The extra carbon in the dialkoxy ring in PProDOT (compare to PEDOT) allows for a symmetric connection to the methacryloyl group. Such a structure may reduce the stress in the final ladder-like brushes, facilitating the formation of a conjugated chain.

### 2.2. Surface-Initiated Atom Transfer Radical Polymerization

**Figure 2:**
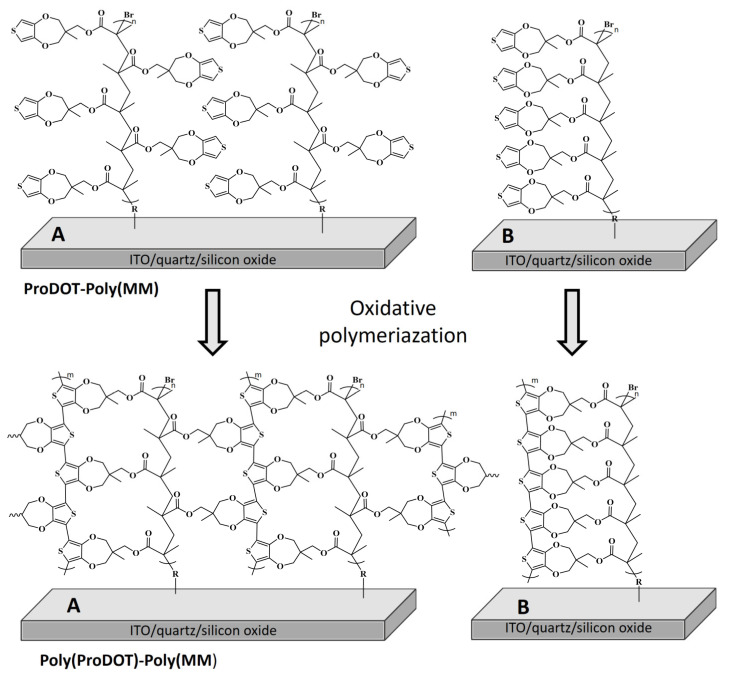
Synthetic route to ladder-like Poly(ProDOT)-Poly(MM) brushes via two reaction pathways: (**A**) intermolecular linking and (**B**) intramolecular linking of ProDOT groups.

ProDOT-Poly(MM) parent brushes were grafted by means of SI-ATRP from different substrates (ITO, silicon oxide wafer and quartz plates; Figure 2). The substrates prior to polymerization were decorated by an ATRP initiator in a two-step process. The first reaction concerned the attachment of APTES molecules to the cleaned surfaces. In the next step, BIB molecules were reacted with the amine groups of APTES, forming a final initiator monolayer, as described elsewhere [40].

The brushes of different thicknesses were obtained by SI-ATRP, realized at 75 °C. The results summarized in Table 1 indicate that the thickness of ProDOT-Poly(MM) brushes increases with polymerization time (Table 1; Figure S11).

**Figure 3:**
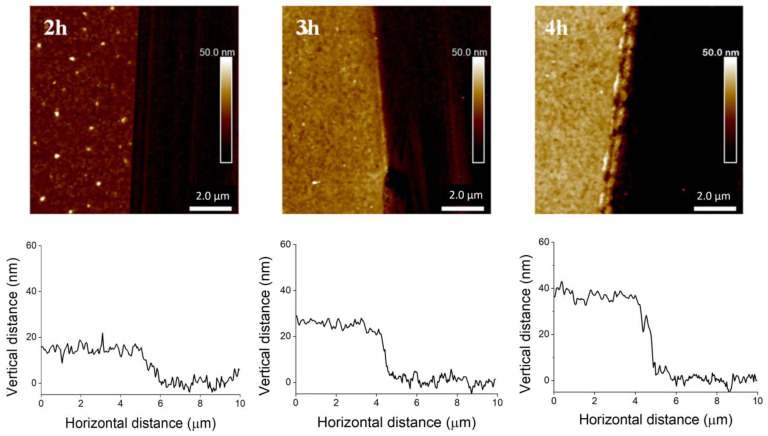
AFM images of ProDOT-Poly(MM) brushes and representative cross-section profiles at the edges showing their thicknesses after 2, 3, and 4 h of polymerization.

**Figure 4:**
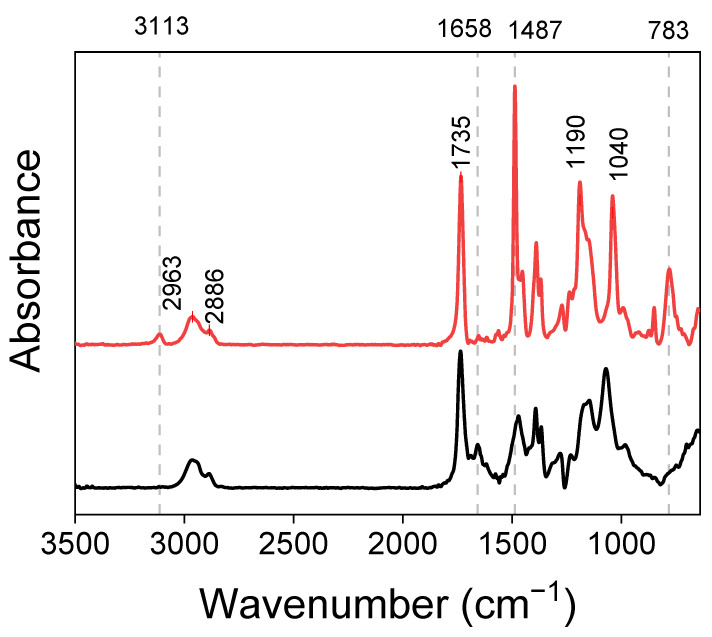
FT-IR spectra of ProDOT-Poly(MM) (red) and Poly(ProDOT)-Poly(MM) (black) brushes.

The successful synthesis of parent brushes after SI-ATRP was confirmed by AFM (Figure 3), grazing angle FT-IR spectroscopy (Figure 4) and XPS spectroscopy (Table 2). The FT-IR spectra show characteristic bands of the thiophene ring: 783 cm^−1^ (α-CH out of plane bending), 1487 cm^−1^ (C=C symmetric stretching vibrations) and 3113 cm^−1^ (α-C-H stretching vibration of hydrogen atoms occurring at positions 2 and 5 of the thiophene ring, next to sulfur) [55]. The IR bands at 1190 and 1040 cm^−1^ were assigned to stretching vibrations in the alkylenedioxy group as well as C-O stretching vibrations in the ester group [22,56,57]. The other peaks at 2963 and 2886 cm^−1^ are characteristic of C-H stretching vibrations in alkyl groups, while the strong band at 1735 cm^−1^ could be assigned to the C=O stretching vibration in the ester group [50].

**Figure 5:**
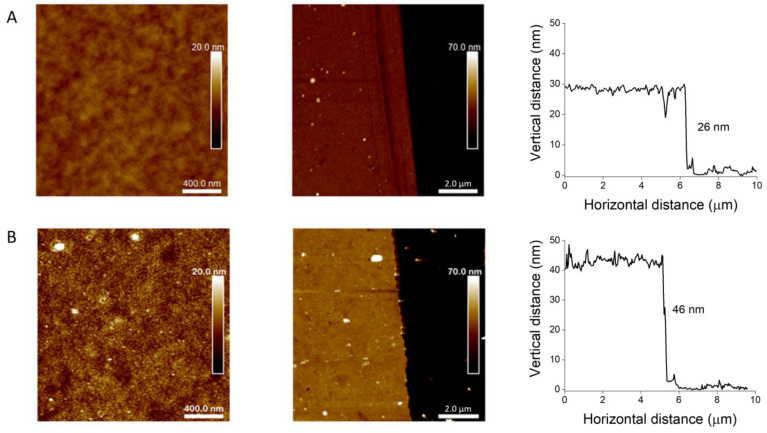
AFM topography images of ProDOT-Poly(MM) (**A**) and Poly(ProDOT)-Poly(MM) (**B**) brushes with corresponding representative cross-section profiles.

AFM measurements confirmed the presence of ProDOT-Poly(MM) brushes and provided information about the thickness of the layer (Figure 3 and Table 1). Moreover, the AFM topography measurement confirmed the uniform coverage of the substrates by the polymer film. The average roughness (R_a_) for the ProDOT-Poly(MM) brushes after SI-ATRP was between 0.6 and 0.8 nm (Figure 5), indicating the creation of a uniform smooth layer.

Initially, the polymerizations were conducted in pure DMF as a solvent. However, satisfactory brush thicknesses were achieved only after quite a long polymerization time in such conditions (Table 1). Moreover, long reaction times resulted in the production of free polymers in the solution (white precipitate), affecting the reproducibility of the formation of thick layers on surfaces. The rate of ATRP depends on several factors, such as: monomer concentration, temperature, the molar ratio of activator to deactivator, ligand type, and solvent [58,59,60]. The increase in temperature resulted in even faster polymer precipitation. The monomer concentration was sufficiently high, while the molar ratio of activator to deactivator (20:1) could not be increased, due to the loss of control over the process. The choice of ligand for the polymerization of thiophene-based bifunctional monomers was optimized in our recent paper [40]. Therefore, we decided to modify the solvent composition by adding 5% water (by volume). The addition of small volumes of water to organic polar solvents was found to substantially accelerate the rate of ATRP [60]. In the case of the ATRP of ProDOT-MM, the utilization of DMF/water (95/5 by volume) solvent mixture greatly enhanced the brushes’ growth, as after only 4 h of polymerization the obtained thicknesses were as high as those after 24 h of ATRP in pure DMF (Table 1). Although unfavorable polymer precipitation after longer reaction times was still observed, the improved polymerization conditions allowed the formation of thick layers using shorter reaction times.

### 2.3. Oxidative Polymerization

In the next step, self-templating polymerization was used to obtain ladder-like conjugated Poly(ProDOT)-Poly(MM) brushes. Pendant ProDOT groups in the parent brushes were subjected to oxidative polymerization with FeCl_3_ that enabled the formation of conjugated polymer chains within the brushes (Figure 2). The FT-IR spectra confirmed the near quantitative conversion of ProDOT groups into conjugated Poly(ProDOT) chains (Figure 4). Indeed, the IR bands characteristic of α-C-H stretching vibrations of hydrogen atoms occurring at positions 2 and 5 of the thiophene ring (3113 cm^−1^) [55] and α-CH out of plane (783 cm^−1^) [22] disappeared after oxidative polymerization (Figure 4). The band at 1487 cm^−1^, assigned to C=C stretching vibrations in the thiophene ring [57,61], significantly decreased and/or was shifted to shorter wavelengths (Figure 4), confirming the formation of conjugated Poly(ProDOT) chains [57,61]. It is worth emphasizing that the completion of oxidative polymerization is also visible to the naked eye as the originally colorless quartz plate coated with unconjugated parent brushes became slightly colored (shades of orange) after the generation of the conjugated chains within the Poly(ProDOT)-Poly(MM).

Differences in topography and thicknesses before and after oxidative polymerization were visualized using AFM. It was found that the thicknesses of the dry brushes after oxidative polymerization increased by 77% (Figure 5). The monomer unit length in saturated polymers was estimated to be 0.26 nm [62], while in case of polythiophene this was around 0.39 nm. Thus, the formation of Poly(ProDOT) chains should force the formed ladder-like chains to adopt a more extended conformation compared to that of ProDOT-Poly(MM) brushes. We observed a similar phenomenon in the case of polythiophene-based brushes [40]. It was expected that the extended conformation of the parent brushes (serving as a template for obtaining conjugated chains) implies the arrangement of pendant groups, leading to the formation of conjugated Poly(ProDOT) chains in the preferred vertical direction with respect to the surface. It is worth emphasizing that such growth may occur according to the intramolecular and also intermolecular mechanisms if the pendant groups from the neighboring chains are close enough to react (see Figure 2). The Density Functional Theory (DFT) calculations indicated that both pathways are feasible for the formation of conjugated Poly(ProDOT) chains (see Supplementary Materials for details). Oxidative polymerization did not significantly change the composition of the brushes, as indicated by the results of XPS measurements, except for residual content of Fe and Cl coming from the oxidative agent (Figure S12; Table 2).

Thus, the obtained conjugated brushes have to present a reduced polymer density and an open (porous) structure (Figure 5), which was recently also proved for the ladder-like poly(3-trimethylsilyl-2-propynyl methacrylate) brushes [63,64]. The roughness value (Ra) increased from 0.6 for ProDOT-Poly(MM) to 1.6 nm for Poly(ProDOT)-Poly(MM), additionally supporting the formation of a stiff and porous polymer layer.

**Figure 6:**
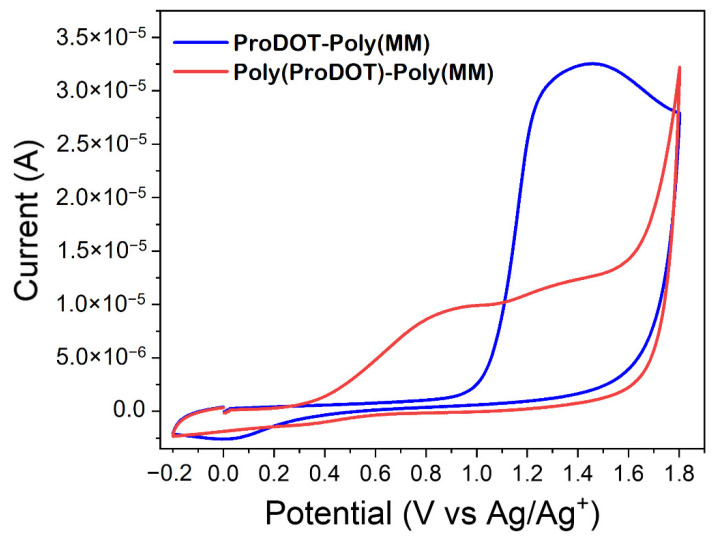
Cyclic voltammograms of ProDOT-Poly(MM) and Poly(ProDOT)-Poly(MM) brushes.

The electrochemical behavior of unconjugated ProDOT-Poly(MM) and conjugated Poly(ProDOT)-Poly(MM) was investigated by cyclic voltammetry, scanning the potential between −0.2 V and +1.8 V (scan rate of 50 mV s^−1^). Cyclic voltammograms were shown in Figure 6. The unconjugated ProDOT-Poly(MM) exhibits one irreversible oxidation peak at $E_{onset}^{ox}=+1.06\;V$ ($E_{max}^{ox}=+1.30\;V$), which may be assigned to the oxidation of thiophene groups. The obtained value roughly correlates with a value from the literature of ProDOT oxidation (+1.2 V), recorded for ProDOT-dienes or ProDOT-based crosslinkers [65,66]. The CV curve for the sample after chemical oxidative polymerization consists of two peaks: one appearing at lower potential ($E_{onset}^{ox}=+0.37\;V$ ($E_{max}^{ox}=+0.9\;V)$) and a residual anodic peak at +1.3 V (Figure 6). The first one may be attributed to the formed conjugated chains and the second one may correspond to some remaining unconjugated ProDOT groups. This is in line with the observation that the first oxidation potential decreased with increasing conjugation lengths [67] and literature data indicating a decreased oxidation potential of Poly(ProDOT) films after electrochemical polymerization as compared to the initial state [65]. Similarly to unoxidized ProDOT, the oxidation process of conjugated brushes is irreversible. Therefore, the performed CV measurements clearly indicate the formation of conjugated Poly(ProDOT)-based chains within the Poly(ProDOT)-Poly(MM) brushes.

**Figure 7:**
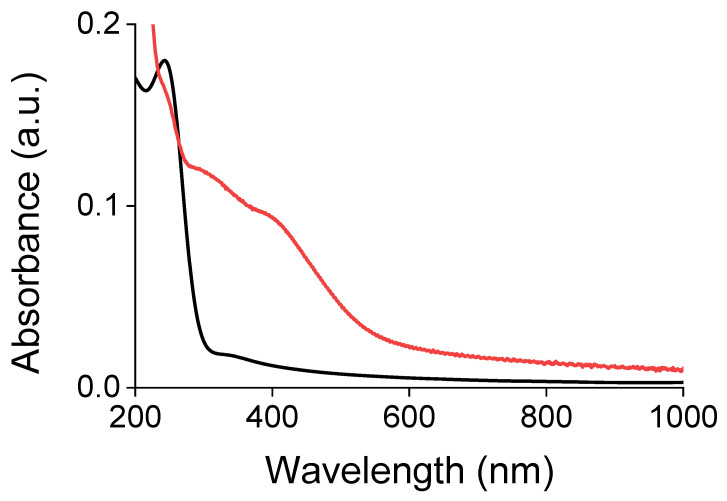
UV-Vis spectrum of the ProDOT-Poly(MM) (black line) and Poly(ProDOT)-Poly(MM) (red line) brush grafted from quartz.

**Figure 8:**
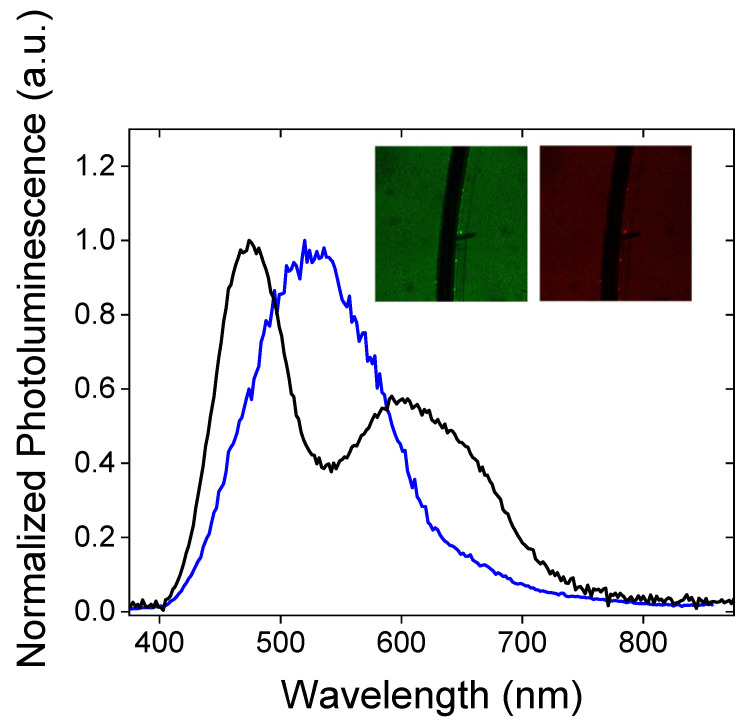
Photoluminescence spectra of Poly(ProDOT)-Poly(MM) brushes grafted from silicon oxide (black) and quartz (blue). Inset: Confocal microphotographs of Poly(ProDOT)-Poly(MM) brush grafted from silicon oxide collected in the green (λ_exc_ = 405 nm) and red channels (λ_exc_ = 488 nm).

The brushes after oxidative polymerization grafted from quartz surface were characterized by UV-Vis spectroscopy (Figure 7) and emission spectroscopy (Figure 8). The UV-Vis absorption spectrum of conjugated Poly(ProDOT)-Poly(MM) exhibits a shoulder between 525 nm and 850 nm and a dominant band with two local maxima at around 310 nm and 400 nm, which are practically absent in the spectrum of the brushes before the conjugation. The deconvolution of the dominant absorption band indicates the presence of at least four peaks (264 nm, 404 nm, 436 nm, and 441 nm) that can be attributed to π-π* electronic transitions of corresponding conjugated chains (Figure S13). The red shoulder of the spectrum shows a band at 722 nm, probably representing π-π stacking and crystallinity within the brushes [68]. It should be stressed, however, that the absorption spectrum interpreted here as a sum of five bands may in fact be composed of a larger number of Gaussian peaks with a narrower full width at half maximum (FWHM) corresponding to oligomers of different lengths. For instance, a broad band with a maximum at 441 nm spans a wide region of UV/UV-vis and completely overlaps with two weaker bands at 404 and 436 nm. Such components may vary in concentration and molar absorptivity, which leads to the formation of rather the band tails, but not the appearance of distinguished maxima. In consequence, a gradual increase in the FWHM of the deconvoluted bands located above 400 nm can be observed.

Referring to a systematic study on β-blocked thiophene oligomers described by Izumi [69], one can roughly assign the abovementioned peaks of the main band to the absorption originated from chains having 2, 4, 6, and 8 mers, respectively [70]. It should be stressed here that the absorption maximum in a dry state (our case) may differ significantly from the one in solution (literature values) due to an increased probability of aggregates forming, as reported by Huddleston et al. for poly(3-methylthiophene) brushes [68]. Therefore, the discrepancies in the absorption maxima of thiophene-based polymer brushes and oligothiophenes in solution can be justified. Furthermore, it is worth remembering that the position of the maximum does not only depend on the number of conjugated mers, but their mutual spatial arrangement as well. The impact may be significant since a lone electron pair located at the oxygen atoms of 3,4-propylenedioxy unit may interact with the lone electron pair of sulfur or oxygen atoms in the neighboring mers, resulting in intrachain twisting (see DFT analysis in Supplementary Material). Izumi [69] and Dai [70] showed that for β-blocked oligothiophenes, the phenomenon of intrachain twisting accounts for the vibronic structure of the absorption and emission bands. On the contrary, linear and coplanar thiophene oligomers are characterized by wide and Gaussian-like bands [71]. Here, the vibronic structure of the UV-Vis spectrum is hidden, probably due to a strong band overlap of conjugated oligomers of different chain lengths, existing in polymer brushes. Nevertheless, the bands located above 300 nm can be applied as an indicator of the presence of conjugated thiophene oligomers.

While the molar absorptivity of the oligomers increases with the increase in their lengths, the contributions of the single thiophene unit is practically independent of the size of the oligomer [69]. For example, the molar absorptivity of four-thiophene-unit-long molecules (45 600 M^−1^⋅cm^−1^) is three times smaller than that of a 12-thiophene-unit-long oligomer (141 000 M^−1^⋅cm^−1^). Thus, it seems that the contribution of the thiophene mers forming longer conjugated chains (six mers and more) in the studied brushes is significant based on the absorption spectrum (Figure 7). Finally, it should also be noted that the length of conjugated oligomers does not reflect the measured length of polymer brushes. It is very likely that the steric effect of the introduced bulky 3,4-propylenedioxy unit may trigger the effective conjugation breaking within the ladder-like brushes, resulting in the formation of conjugated sections shorter than the contour length of the macromolecules (Figure S14).

Photoluminescence spectra of the Poly(ProDOT)-Poly(MM) brushes grafted from quartz show a dominant band centered at 525 nm and a red-shifted shoulder between 600 nm and 800 nm (Figure 8). Photoluminescence is very sensitive to the presence of impurities and hence the emission signal can be distorted by them. However, the measured photoluminescence excitation spectra (see Figure S15) showed a good correlation of the position of excitation bands with the absorption signal, indicating that the observed photoluminescence originated from the studied conjugated chains. Similarly to the analysis of the absorption spectrum, we used the emission maxima of the related oligomers (4-, 6-, and 12-thiophene-unit-long β-blocking oligothiophenes [69]) and reconstructed the shape of photoluminescence spectra by fitting Gaussian function. The results of fitting are depicted in Figure S16. From the fitting results, one can conclude that the bands with maxima at 453 nm and 499 nm may be assigned to the chains having four thiophene-based mers (literature values: 453.2 and 481.6 [69]). The band at 536 nm probably corresponds to the emission of six effectively conjugated mers; the literature spectrum consists of two bands at 514.4 nm and 551.4 nm, and the maximum of the deconvoluted band correlates with an arithmetic mean of the two mentioned maxima. However, one cannot exclude the origin of this maximum from the segments of longer conjugation; PEDOT octamers show an emission maximum at 510 nm [70]. The last pair of bands at 568 nm and 596 nm may be attributed to 12 and/or more thiophene units in conjugated chains [69,70]. The analysis of the FWHM of photoluminescence spectra is rather problematic since the deconvoluted bands should be considered in pairs. Nevertheless, one can notice an increase in FWHM while going to longer wavelengths (Figures S16 and S17) as the number of available conformations will be larger for longer conjugations.

Interestingly, the emission spectrum of Poly(ProDOT)-Poly(MM) brushes grafted from silicon oxide demonstrates two well-separated luminescence maxima (Figure 8). The deconvolution analysis revealed the presence of at least six individual bands at 455 nm, 482 nm, 515 nm, 553 nm, 588 nm, and 636 nm that can be assigned to the corresponding chains (Figure S17). As previously shown, four conjugated thiophene units are presumably responsible for the emissions at 455 nm and 482 nm, while the pair of bands at 515 nm and 553 may be attributed to the emission of 6-10 thiophene units. In the case of broadband at 588 nm and 636 nm, such analysis may be difficult since the emission maxima of oligomers longer than 10 thiophene units do not differ much from each other. Hence, this spectral region may reflect the emission of the large spectrum of long conjugates (longer than 10 thiophene subunits) distributed within the layer of polymer brushes. Finally, it is pertinent to note that this spectral assignment (mostly based on the results obtained for thiophene in solution) does not consider the effect of the surface and the solid state of the investigated system. Therefore, one can expect anomalies in the position of bands, full width at half maximum or the presence of aggregates, which are rather unlikely to be observed in diluted polythiophene-based solutions.

**Figure 9:**
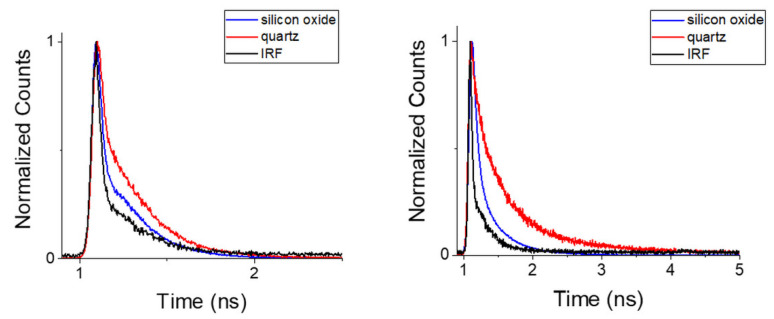
Photoluminescence decays collected for Poly(ProDOT)-Poly(MM) brushes grafted from quartz (red) and from silicon oxide (blue), together with the instrument response function (IRF, black). Left: decays monitored at blue region of emission band (λ < 492nm); right: those monitored at red region of emission band (595 nm < λ < 665 nm).

One can notice a significant impact of the substrate surface on the distribution of the effective conjugation lengths in the brushes (Figure 8). It is supposed that this effect is related to surface roughness, which may imply variations of both chain conformations and mutual interaction. In our case, AFM measurements indicate an enlarged roughness of quartz as compared to silicon oxide surface (Figure S18). It seems that the smoother surface of silicon oxide may keep the chains of polymer brushes straighter, while the roughness of quartz may contribute to a higher probability of intermolecular bonding, resulting in the formation of a more rigid structure, and therefore preventing dynamic (collisional) quenching. This finding is also reflected in the photoluminescence lifetimes (monitored at 630/69 nm; see the decays in Figure 9), which are listed in Table S3. Straight polymer brushes exhibit very short luminescence lifetimes, presumably because of the fast internal conversion related to chain motions or electron transfer-caused structural reorganization (τ = 180 ps).

Both the abovementioned processes are less feasible for rigid structures observed for the polymer brushes grafted from quartz. Consequently, the luminescence lifetimes (τ = 630 ps) monitored at 630/69 nm are significantly longer. Interestingly, short conjugated oligomers (four units) exhibit short luminescence lifetimes (180 ps on quartz; 167 ps on silicon oxide), which do not depend on the substrate surface (Figure 9).

**Figure 10:**
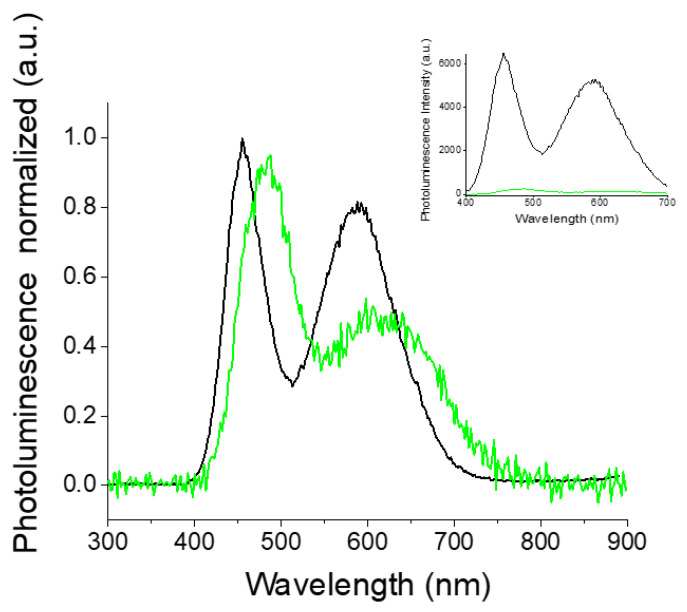
Normalized photoluminescence spectra of Poly(ProDOT)-Poly(MM) brushes grafted from silicon oxide: 20 nm (black) and 37 nm (green). Inset: non-normalized photoluminescence spectra of the mentioned materials presenting the differences in emissive properties.

This unusual emission behavior of the brushes grafted from silicon oxide inspired us to study the impact of the height of the conjugated polymer brushes and temperature on their photophysical properties. It turned out that the height of the brushes affects both the position of the bands and the emission intensity as well (Figure 10).

The brushes with a height of 37 nm display two emission bands red shifted by about 31 nm as compared to the thinner ones (20 nm of height). This slight red shift may be interpreted in the context of an increased number of longer conjugated chains (longer than 4 mers [15]), which may be related to a larger ability of thicker polymer brushes to their formation. However, thicker polymer brushes also have a tendency to form aggregates. Such structures may indeed be possible since the total photoluminescence intensity drops dramatically when the height of the brushes increases. The observed phenomenon is probably due to the aggregation-caused quenching process [72]. Finally, our temperature-dependent luminescence studies revealed no substantial changes in the emission spectra, indicating that the molecular structure adopted by the brushes in a dry state is rather insensitive to changes in temperature. Typical oxygen sensitivity values of the studied polymer brushes were observed. However, this effect is more pronounced for long-lived excited polymer brushes grafted from quartz (Figure S19).

### 2.4. Stability of the Brushes

We have previously shown that polythiophene-based brushes demonstrated more red-shifted absorption maxima when oxidative polymerization was performed in the nitromethane [40]. However, besides the formation of conjugated chains with longer conjugation lengths, the brushes were doped by FeCl_3_ and required dedoping after synthesis. The dedoping process decreased their stability, leading to an increased content of oxygen, as indicated by XPS and the appearance of carbonyl-like bands in the FT-IR spectra [40]. Therefore, in this report, ProDOT-Poly(MM) brushes were polymerized in dry chloroform, which enabled the formation of the conjugated chains in their native (undoped) form. In the case of previously reported poly(3-methylthienyl methacrylate) (PMTM) brushes as well as polythiophene polymers, chain degradation starts from the β-carbons [40]. In ProDOT-Poly(MM) brushes, the positions of β-carbons in the thiophene rings are blocked, which may bring the benefits of improved stability, as well as a stiffer and more ordered conformation.

**Figure 11:**
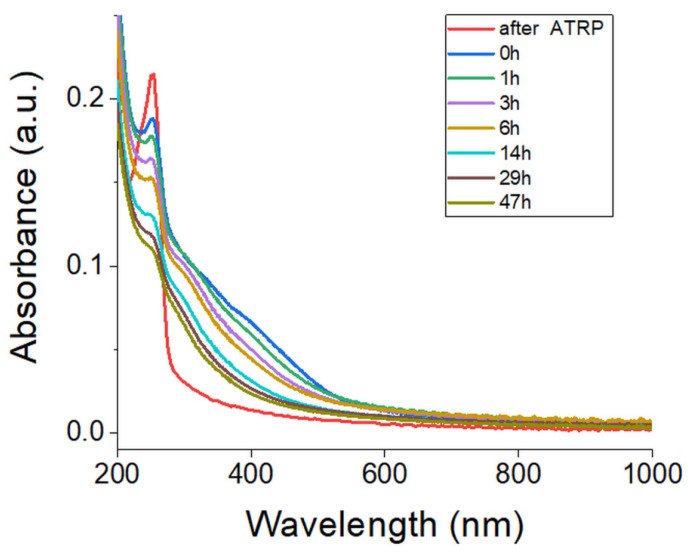
UV-Vis spectra of the Poly(ProDOT)-Poly(MM) brushes grafted from quartz (25 nm thick) at different times after irradiation under ambient conditions. The spectrum of nonconjugated ProDOT)-Poly(MM) brushes (after ATRP) is shown for comparison.

The time-dependent stability of the ladder-like Poly(ProDOT)-Poly(MM) brushes was investigated by means of UV-Vis (Figure 11) and FT-IR spectroscopy (Figure S20). The sample was stored for the entire period with exposition to room light and air at room temperature. After 3 days of exposure, we did not notice any changes in the FT-IR spectra of the brushes. Over the 81 days, the measurements revealed only minor intensity changes of the band at around 1650 cm^−1^, which is an indicator of the oxidation of the conjugated chains [40] and a slight increase in the band at 1735 and 1170 cm^−1^ (Figure S20). The performed tests revealed the higher stability of the Poly(ProDOT)-Poly(MM) brushes against oxidation compared to the PMTM brushes, which exhibited more noticeable oxidation after a few days of storage [40].

The stability of the ladder-like Poly(ProDOT)-Poly(MM) brushes was also evaluated under exposure to standardized solar light (AM 1.5 G) of 100 mW/cm^2^. The experiment was conducted under ambient conditions (in the air, humidity 40%) for the brushes grafted from quartz surfaces. UV-Vis spectra were measured before and after illumination (Figure 11).

**Figure 12:**
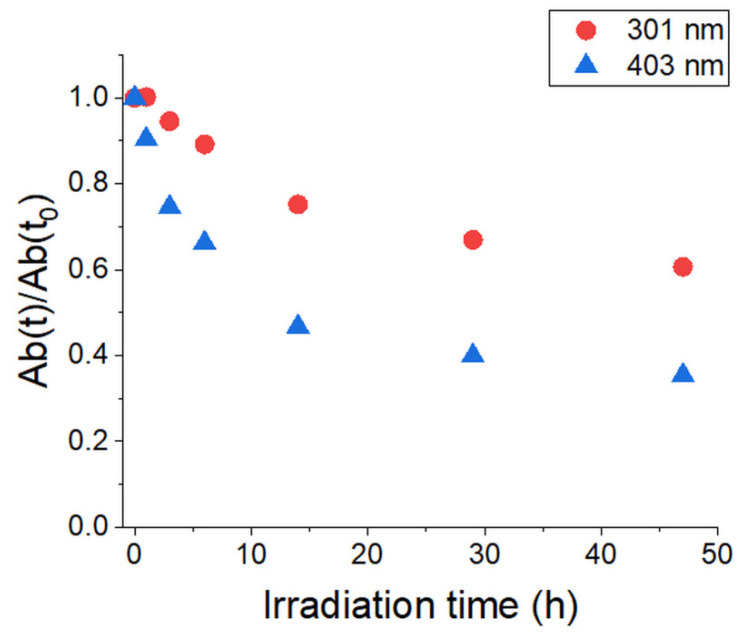
Normalized UV-Vis absorption of Poly(ProDOT)-Poly(MM) at 301 nm and 403 nm during AM 1.5 G light irradiation.

The intensity and shape changes of the band at 400 nm were observed after irradiation with standardized solar light. After 47 h of irradiation, the intensity of the band at 400 nm substantially decreased for the studied sample. Just like in the case of polythiophene derivatives, the visible absorption intensities decreased continuously during the irradiation and the absorption maxima were blue shifted [73]. These results suggest a reduction in the conjugation length of the Poly(ProDOT)-Poly(MM) brushes [74]. However, despite the four times lower thickness of polymer brushes with respect to the films of polythiophene derivatives obtained by Aoyama et al., the ratio of the absorbance after irradiation to the initial absorbance for Poly(ProDOT)-Poly(MM) brushes is comparable to the best-performing polymer presented by Aoyama et al. (Figure 12) [73]. These experiments prove the high stability of the Poly(ProDOT)-Poly(MM) brushes, which is essential for their future applications in optoelectronic devices.

## 3. Materials and Methods

### 3.1. Materials

3,4-Dimethoxythiophene (>97%), p-Toluenesulfonic acid monohydrate (p-TSA, 98%), and magnesium sulfate (anhydrous 99.5%) were all purchased from Fluorochem (Derbyshire, UK). Triethylamine (TEA, >99.5%), 3-aminopropyl-trimethoxysilane (APTES, 98%), α-bromoisobutyryl bromide (BIB, 98%), 1,1,4,7,10,10-hexamethyltriethylenetetramine (HMTETA, 97%), copper(II) bromide (99.999%), copper(I) bromide (99.999%), and DMF (>99.9%) were purchased from Sigma-Aldrich (St Louis, MO, USA). Dichloromethane (DCM, p.a.), chloroform (p.a.), toluene (p.a.), dimethylformamide (DMF, p.a.), tetrahydrofuran (THF, p.a.), methanol (p.a.), ethanol (99.9%), hydrogen peroxide (30% p.a.), and ammonia solution (30% p.a.) were all purchased from Chempur (Piekary Slaskie, Poland). Hydrochloric acid (35–38% p.a.) was obtained from POCH S. A. (Gliwice, Poland). 1,1,1-Tris(hydroxymethyl)ethane (>98%) was obtained from TCI Co. (Tokyo, Japan). Iron(III) chloride (99.5%) was purchased from Lab-Scan (Gliwice, Poland). Distilled chloroform and methanol used for sample purification after oxidative polymerization were additionally dried under molecular sieves (0.3 nm, Merck, Darmstadt, Germany) before use. The rest of the chemicals were used as received.

### 3.2. Methods

The FT-IR spectra of PProDOT-based brushes grafted onto the ITO surface were recorded using a Nicolet iS10 FT-IR spectrometer with a grazing-angle reflectance accessory (at an incident angle of 80°). The FT-IR spectrum of the monomer was captured and averaged over 128 scans using the same spectrometer equipped with an ATR accessory. All spectra were baseline corrected. The NMR spectrum of the monomer was obtained using an Avance III HD 400 MHz spectrometer (Bruker). LCMS analysis was performed employing an analytical set of an ultra-high performance liquid chromatography from Dionex UltiMate 3000 with a UV-Vis detector coupled with a high-resolution mass spectrometer with a time-of-flight analyzer (ESIQTOF)—Bruker Impact II. A gradient method was used (column: Gemini NX—C18, 150 × 3.0, 3 μm 110 A; phase A: H_2_O + formic acid (0.1%); phase B: methanol + formic acid (0.1%)). Only the positive ionization method yielded results. Confocal micrographs were collected using an inverted microscope, Nikon Ti-E, with objective Plan Apo 100×/1.4 Oil DIC and confocal system Nikon A1. Atomic Force Microscopy (AFM) images were obtained using a Dimension Icon AFM (Bruker, Santa Barbara, CA, USA) working in the PeakForce and QNM^®^ modes. AFM probes with standard silicon cantilevers (a nominal spring constant of 0.4 N·m^−1^) were used for measurements. The dry thicknesses of the brushes were determined in air using AFM measurements at the edges of a scratch formed in the brush layer using needles or tweezers. The thickness was measured in several locations, averaged and presented as the arithmetic mean ± standard deviation.

The UV-Vis spectra of the brushes were recorded on a Varian Cary 50 UV-Vis spectrophotometer in transmittance mode. The UV-Vis spectrum of the ethanolic monomer solution was measured by a UV-Vis Cary 60 Agilent spectrophotometer. XPS measurements were performed with a Prevac photoelectron spectrometer (Rogów, Poland) equipped with a hemispherical analyzer VG SCIENTA R3000. The spectra were recorded using a monochromatized aluminum source, Al Kα (E = 1486.6 eV). The surface composition of polymer brushes was studied based on the areas and binding energies of C 1s, O 1s, S 2p, Fe 2p, and Cl 2p. The spectra were fitted using the CasaXPS software (Casa Software Ltd., Teignmouth, UK).

The photoluminescence spectra and lifetime data were measured using a home-built wide-field microscope. Briefly, a 405nm pulsed laser (PicoQuant LDH-D-C-405) was focused on the back aperture of an LucplanFI 40X objective (Olympus) to achieve a wide-field illumination of the sample. After excitation, the photoluminescence from the sample was collected by the same objective and subsequently guided toward the detectors. For the photoluminescence spectra, a slit was used to limit the region from which fluorescence is collected from and a diffraction grating was placed in front of the camera allowing us to record the spectrum of the light going through the slit on the EMCCD camera (Princeton Instruments, ProEM). For the photoluminescence lifetime, the detected signal was guided through a pinhole toward a single photon avalanche diode (MPD, PDM series). The pinhole was used to determine the exact position from where the lifetime was measured from. We used a 492 SP short pass filter and a 638-69 BP band pass filter to measure different parts of the spectrum separately.

Due to the presence of the complex photoluminescence kinetics of the polymer brushes, the decays were fitted by a sum of exponentials:

It=∑iAie−tτi.

The obtained results were used to calculated intensity-weighted mean lifetimes with the following formula [75]:

$$\tau_{I}=\frac{\sum_{i}A_{i}\tau_{i}^{2}}{\sum_{i}A_{i}\tau_{i}}.$$

The peak deconvolution program implemented in Origin 2019 software was employed for the spectral decomposition. Both absorption and emission spectra were fitted to a sum of individual Gaussian functions. No constraints were used in terms of the spectral width of the fitted Gaussian curves. In the course of fitting, the lowest possible number of Gaussians were applied to reproduce the shape of the spectrum and simultaneously to yield the best R^2^ parameter.

Cyclic voltammetry (CV) measurements were carried out using an Autolab PGSTAT204 potentiostat. A platinum wire (ø = 1 mm) and nonaqueous screw-type electrode Ag/Ag+ (0.1 M tetrabutylammonium perchlorate) were used as counter and reference electrodes, respectively. An ITO plate modified with polymer brushes was employed as a working electrode. A 0.1 M solution of tetrabutylammonium perchlorate (Aldrich, ≥99.0%) in acetonitrile (Aldrich, 99.8% HPLC grade) was used as an electrolyte. To remove residual oxygen, solutions were purged with argon. Redox potentials were taken from the onset of the first oxidation potential.

Density functional theory (DFT) calculations were carried out with the Gaussian16 package [76]. For geometry optimizations in the ground state, the global hybrid B3LYP [77,78,79] exchange-correlation functional and a Dunning correlation-consistent, polarized valence [80], double-ζ basis set, cc-pVDZ, were employed. For such a large and flexible molecular system, loose convergence criteria were applied during the calculations.

### 3.3. Synthesis of (3-Methyl-2,4-dihydrothieno[3,4-b][1,4]dioxepin-3-yl)methanol (ProDOT-OH)

3,4-Dimethoxythiophene (3.55 g, 24.6 mmol), 1,1,1-tris(hydroxymethyl)ethane (3.8 g, 32 mmol), and p-TSA monohydrate (0.4 g, 2.4 mmol) catalyst were added to a round bottom flask with a magnetic stirring bar and distilled toluene (300 mL) inside. The reaction mixture was heated and stirred at 110 °C for one day. Dean–Stark apparatus was used to allow water to be removed. The reaction mixture was cooled to room temperature and then the black precipitate (side product) was filtered off. Toluene was evaporated from the filtrated solution under reduced pressure using a rotary evaporator. Then, the mixture was dissolved in methylene chloride and washed three times with NaCl (aq) and once with water. The organic phase was extracted by DCM and dried over MgSO_4_. After solvent evaporation, the obtained greenish residue was purified by column chromatography (hexane/ethyl acetate = 4/1), which resulted in obtaining 3.9 g (yield 80%) of viscous yellowish oil. ^1^H NMR (400 MHz, CDCl_3_, δ): 0.95 (s, -CH_3_, 3H), 1.64 (s, -OH, 1H), 3.73 (d, -OCH_2_, 4H), 4.06 (s, -CH_2_-, 1H), 4.09 (s, -CH_2_-, 1H), and 6.48 (s, thiophene ring, 2H) (Figure S1). ^13^C NMR (400 MHz, CDCl_3_, δ): 149.61, 105.62, 76.48, 65.76, 43.73, and 16.97 ppm (Figure S2). IR (ATR, cm^−1^): 3292 (O-H), 3113, 3101 (C-H), 2941 (C-H), 2867 (C-H), 1487, 1449, 1385 (C=C), 1367 (C-C), 1186 (C-H) 1065, 1045, 1022 (C-O-C), 851, 781, and 758 (C-H) (Figure S3 and Table S1). UV-VIS absorption maximum: 251 nm (Figure S4). LCMS (Figure S5, positive ion mode): m/z found for [M+H]^+^ amounts, 201.058.

### 3.4. Synthesis of (3-methyl-2,4-dihydrothieno[3,4-b][1,4]dioxepin-3-yl)methyl 2-methylprop-2-enoate (ProDOT-MM)

ProDOT-OH (3.78 g, 18.8 mmol), triethylamine (3.55 g, 35 mmol), and 100 mL of dry dichloromethane were mixed under argon in the flask stopped with a rubber septum and placed on the magnetic stirrer. The distilled methacryloyl chloride (2.7 mL, 25.8 mmol) was added dropwise to the reaction mixture and left to react for two days. The final reaction solution was washed with large amounts of 1 M HCl (aq), NH_4_Cl (aq) and water. The organic phase was extracted with DCM and dried over MgSO_4_. After this, the solvent was evaporated and the obtained residue was purified by column chromatography (hexane/ethyl acetate = 4/1), giving 4.13 g (81%) of the final product (white solid). ^1^H NMR (400 MHz, CDCl_3_, δ): 1.01 (s, -CH_3_, 3H), 1.96 (s, CH_3_, 3H), 3.78 (d, -OCH_2_, 2H), 4.04 (d, -OCH_2_, 2H), 4.26 (s, -CH_2_-, 2H), 5.59 (m, =CH_2_, 1H), 6.12 (m, =CH_2_, 1H), and 6.49 (s, thiophen ring, 2H) (Figure S6). ^13^C NMR (400 MHz, CDCl_3_, δ): 167.08, 149.55, 136.06, 125.80, 105.72, 76.25, 66.60, 42.75, 18.30, and 17.24 ppm (Figure S7). IR (ATR, cm^−1^): 797 (C-H), 1044 (C-O-C), 1182 (C-H), 1379 (C-C), 1403, 1450, 1489 (C=C), 1639 (C=C), 1712 (C=O), 2925, 2955, 2970 (C-H), 2941 (C-H), 3102, and 3112 (C-H) (Figure S8 and Table S2). UV-VIS absorption maximum: 250 nm (Figure S9). LCMS (Figure S10, positive ion mode): m/z found for [M+H]^+^ amounts, 269.084.

### 3.5. SI-ATRP of ProDOT-MM

The ATRP initiator was grafted in a two-step synthesis either on ITO, quartz or silicon oxide plates according to the previously reported procedure [40], by depositing APTES followed by surface reaction with BIB. Beforehand, the plates were cleaned by rinsing with ethanol and dried under a stream of argon. The plates were then put into the cleaning mixtures (ITO plates were put into the 1:1 mixture of 30% ammonia solution with 30% hydrogen peroxide at 50 °C for 1 h, while quartz or silicon oxide plates were put into the 3:1 mixture of 96% sulfuric acid with 30% hydrogen peroxide at room temperature for 1 h). Next, the plates were rinsed with copious amounts of water, THF, and toluene. Then, they were dried in the stream of argon. Finally, the supports were cleaned by a hand-held plasma cleaner for one minute.

The reaction system used in ATRP polymerization was composed of 4 glass vessels stopped with rubber septa and connected with each other via cannulas. In the first vessel, DMF was placed to saturate the reaction system with its vapors. In the next vessel, DMF (0.285 mL), water (0.015 mL), CuBr_2_, (0.056 mg, 0,000252 mmol), HMTETA (5.24 µL, 0.019 mmol) and ProDOT-MM monomer (134.16 mg, 0.5 mmol) were mixed. CuBr (0.75 mg, 0.005 mmol) and a magnetic stirring bar were placed in the third vessel. Finally, the initiator modified-substrate was placed inside a glass cuvette in the last vessel (optical path length—2 mm; max volume—0.7 mL). The molar ratio of the reagents was as follows: CuBr_2_ (1):CuBr (20):HMTETA (77):ProDOT-MM (2000). The whole reaction system was purged with argon for 15 min and then the solution from the second vessel was moved to the third vessel under argon via a cannula. After the dissolution of CuBr, the contents of the third vessel were transferred under argon to the polymerization vessel (with initiator-modified substrate) immersed in a preheated oil bath (75 °C). The ATRP was conducted for a given time at 75 °C to achieve the desired thickness of the layer. After completion, the polymerization was stopped by opening the vessel in air. The plate with grafted ProDOT-Poly(MM) brushes was cleaned by rinsing and sonicating in organic solvents: DMF, THF, and toluene. Finally, the plate was dried in argon.

### 3.6. Self-Templating Oxidative Polymerization of ProDOT-Poly(MM) Brushes

ITO, quartz, or silicon oxide plate with surface-grafted ProDOT-Poly(MM) brushes was immobilized on the specially designed glass tripod placed in an amber glass vessel. A total of 20 mg of anhydrous FeCl_3_ and a small magnetic stirring bar were then added. In another vessel, 20 mL of freshly distilled chloroform was poured. Both vessels were stopped by rubber septa, connected by a cannula, and placed in an ice bath (0 °C). After purging with argon (20 min), chloroform was transferred to the vessel with the plate and gently stirred at 0 °C for 1 h in dark. After this, the reaction mixture was placed in the fridge, adjusted at 4 °C and left to react for 23 h. Finally, the reaction was heated to room temperature and left for 24 h. After completion, the sample was cleaned by rinsing with chloroform and methanol under argon in a glove box. The brushes after this step are abbreviated as Poly(ProDOT)-Poly(MM).

## 4. Conclusions

We present here, for the first time, a comprehensive report on the synthesis of PProDOT-based brushes via surface-initiated chain growth polymerization. The synthetic route based on a two-step ST-SIP method led to new ladder-like Poly(ProDOT)-Poly(MM) brushes with improved (photo)stability as compared to polythiophene-based brushes. At first, the SI-ATRP of the bifunctional ProDOT-MM monomer enabled the synthesis of parent ProDOT-Poly(MM) brushes with pendant ProDOT groups. These groups were subsequently polymerized in the next step using oxidative polymerization with FeCl_3_, leading to the final Poly(ProDOT)-Poly(MM) brushes with conjugated Poly(ProDOT) chains in a ladder-like architecture. The application of SI-ATRP allowed the formation of dense parent brushes with dry thicknesses in the range of 15–40 nm. The rate of polymerization was controlled by varying the solvent composition. The applied conditions of oxidative polymerization allowed the quantitative conversion of ProDOT groups into conjugated PProDOT chains, which resulted in substantial increase in dry brush thickness. Our photophysical analysis (UV-VIS absorption, photoluminescence, and lifetimes) provided information about the population of macromolecular segments having various effective conjugation lengths within the brushes. Conjugated Poly(ProDOT)-Poly(MM) brushes exhibited very good stability in ambient conditions, as revealed from the FT-IR measurements. The high stability was further confirmed by photodegradation measurements performed using standardized solar light (AM 1.5 G) of 100 mW/cm^2^. The abovementioned results indicate that the ladder-like architecture of conjugated Poly(ProDOT)-Poly(MM) brushes might be advantageous in the case of fabricating highly ordered and stable molecular wires based on hardly processable conjugated polymers for potential applications in, e.g., sensors and nanoelectronics.

## Figures and Tables

**Table 1:** Polymerization conditions, time and thickness of the ProDOT-Poly(MM) brushes grafted from ITO.

Solvent Composition: DMF/Water ^a^	Time of Polymerization	Thickness of ProDOT-Poly(MM) Brushes
95/5	2 h	14 ± 2 nm
95/5	3 h	24 ± 1 nm
95/5	4 h	36 ± 2 nm
100/0	10 h	14 ± 2 nm
100/0	22 h	39 ± 4 nm

^a^ By volume, the molar ratio of reagents: CuBr_2_ (1):CuBr (20):HMTETA (77):ProDOT-MM (2000).

**Table 2:** Atomic concentration determined by XPS for ProDOT-Poly(MM) and Poly(ProDOT)-Poly(MM) brushes grafted from quartz.

	Theoretical Atomic Concentration (%)	Atomic Concentration of ProDOT-Poly(MM) (%)	Atomic Concentration of Poly(ProDOT)-Poly(MM) (%)
C	72.2	69.8	69.0
O	22.2	23.9	24.8
S	5.6	6.3	6.0
Fe	-	-	0.1
Cl	-	-	0.1

## Data Availability

Data are contained within the article or Supplementary Materials.

## Supplementary Materials

The following supporting information can be downloaded at: https://www.mdpi.com/article/10.3390/ijms23115886/s1.
