# Supplementary material for: Ladder-like Polymer Brushes Containing Conjugated Poly(Propylenedioxythiophene) Chains
Source: Int J Mol Sci. 2022 May 24;23(11):5886. doi: 10.3390/ijms23115886 (PMC9180196; doi:10.3390/ijms23115886)
Supplement: Supplementary file 1 [file ijms-23-05886-s001.zip › ijms-1681623-supplementary.pdf]

Ladder-like polymer brushes containing conjugated poly(propylenedioxythiophene) chains

Gabriela Grześ[1], Karol Wolski[1,], Tomasz Uchacz[1], Justyna Bała[1], Boris Louis[2,3], Ivan G. Scheblykin[2], Szczepan Zapotoczny[1,*]*

[1] Faculty of Chemistry, Jagiellonian University, Gronostajowa 2, 30-387 Krakow, Poland

[2] Division of Chemical Physics and NanoLund, Lund University, Lund 22100, Sweden

[3] Molecular Imaging and Photonics, Department of Chemistry, KU Leuven, Celestijnenlaan 200F, 3001 Leuven, Belgium

Characterization of (3-methyl-2,4-dihydrothieno[3,4-b][1,4]dioxepin-3-yl)methanol (ProDOT-OH)

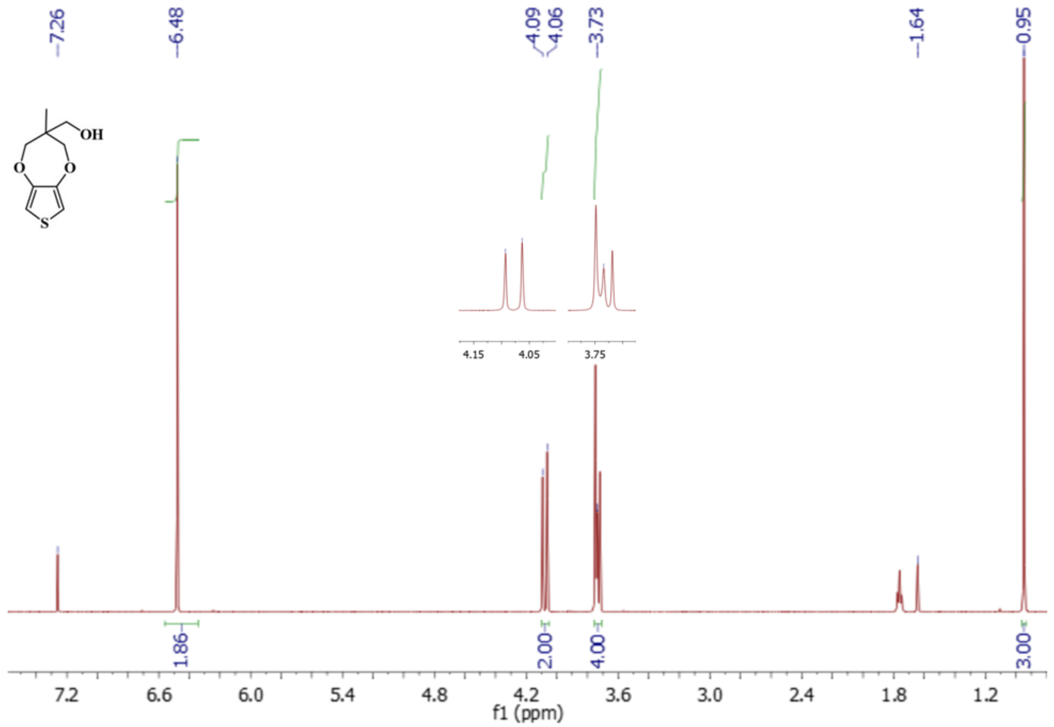

Figure S1. ^{1}H NMR spectrum of ProDOT-OH.

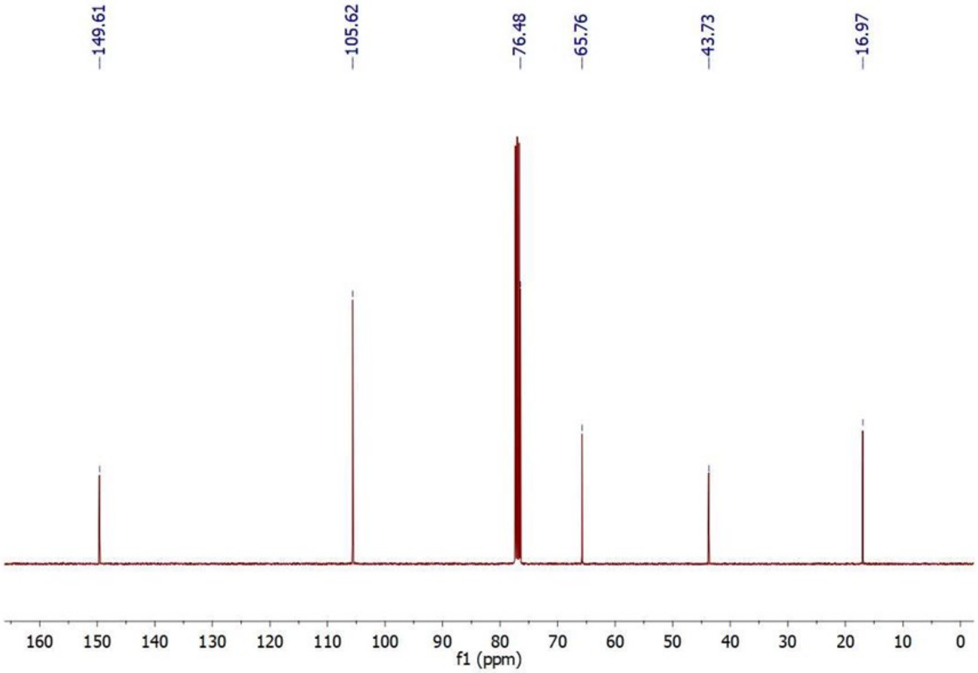

Figure S2. ^{13}C NMR spectrum of ProDOT-OH.

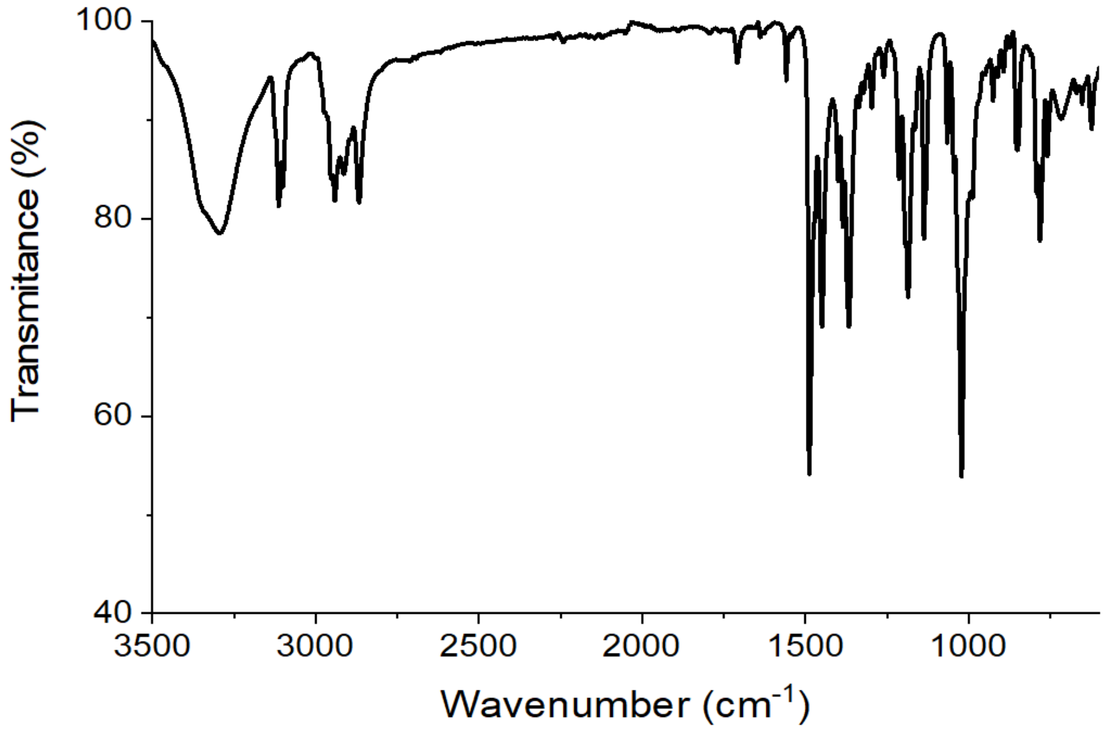

Figure S3. FT-IR spectrum of ProDOT-OH monomer.

Table S1. Assignments of major bands in FT-IR spectrum presented in *Figure S3* [1].

Band position (cm^{-1})	Assignments and Notes
3293	O-H stretch from end O-H of ProDOT-OH
3113	C-H stretching from 2,5 position of thiophene group
3101	
2941	C-H stretching from ProDOT ring
2867	C-H stretching from ProDOT ring and alkyl chains
1488	C=C stretching from thiophenes
1449	
1385	
1367	C-C stretching from cyclopropylene group of ProDOT
1186	C-H in plane

1065	C-O-C stretching from ProDOT ring
1045	
1022	
851	C-H out of plane
781	
758	

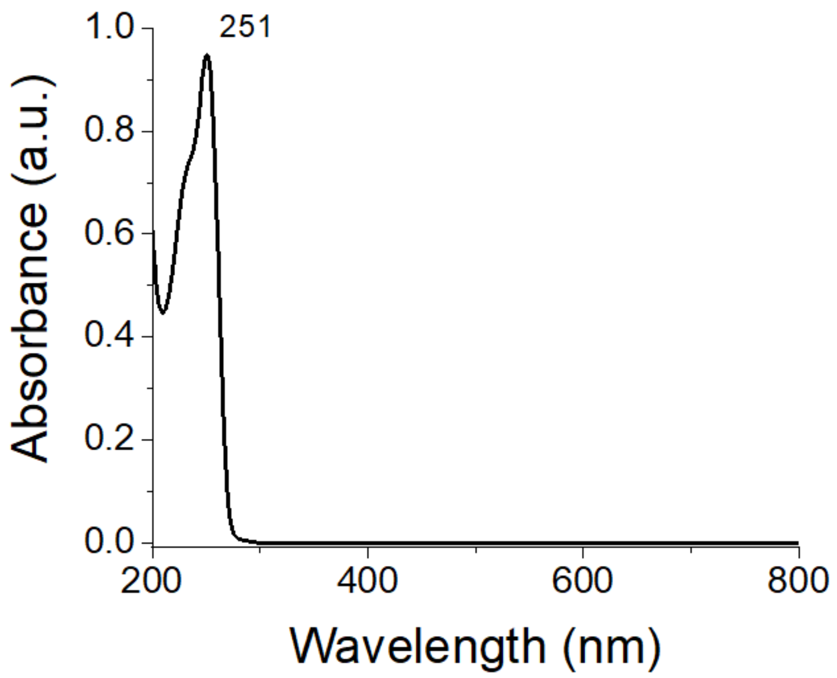

Figure S4. UV-Vis spectrum of ProDOT-OH.

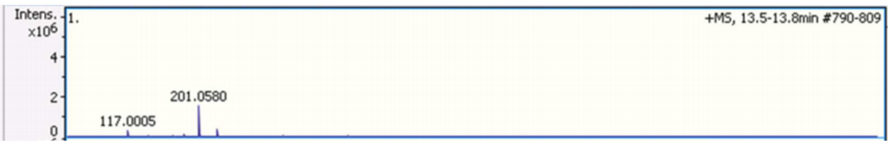

Figure S5. LCMS spectrum of ProDOT-OH.

Characterization of (3-methyl-2,4-dihydrothieno[3,4-b][1,4]dioxepin-3-yl)methyl 2-methylprop-2-enoate (ProDOT-MM)

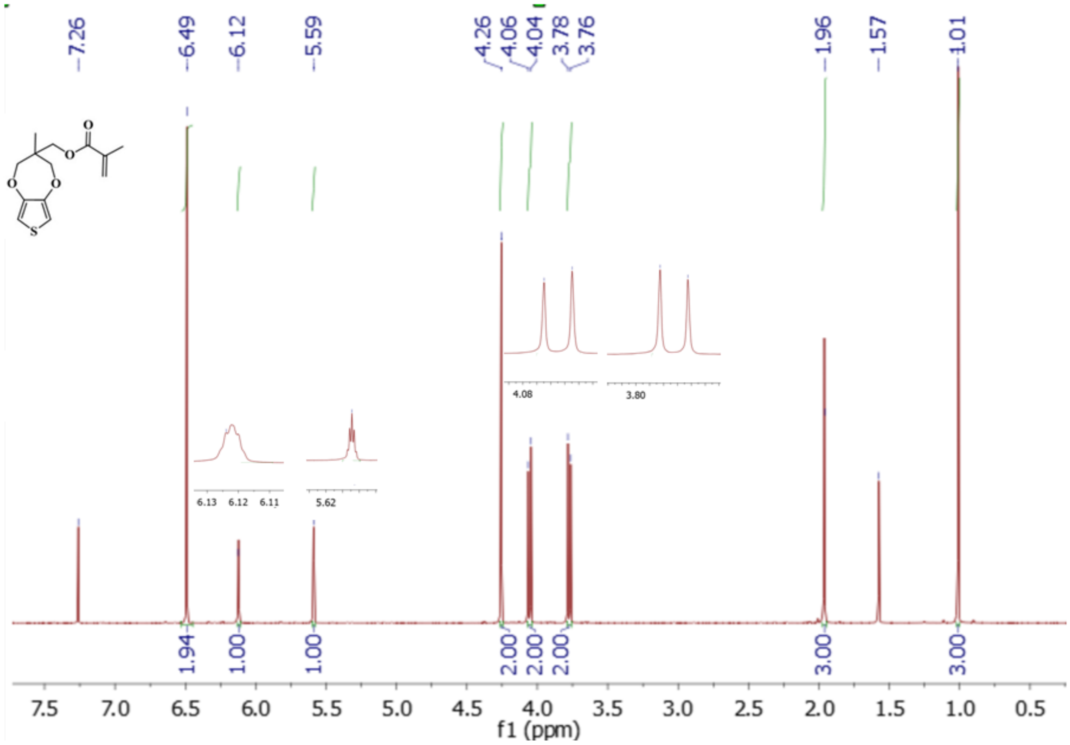

Figure S6. ^{1}H NMR spectrum of ProDOT-MM monomer.

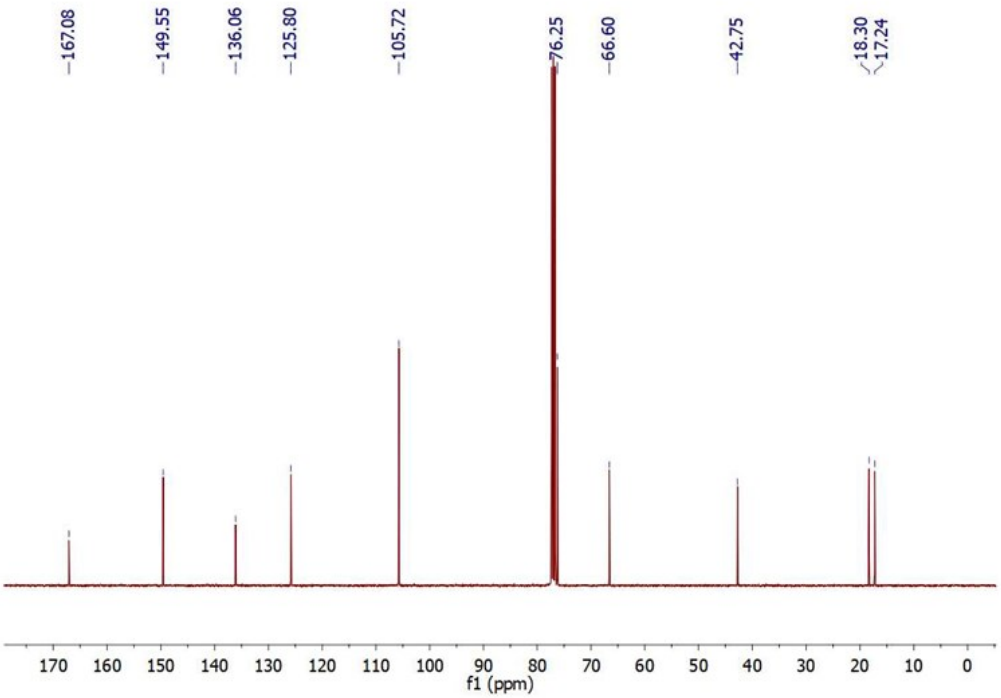

Figure S7. ^{13}C NMR spectrum of ProDOT-MM monomer.

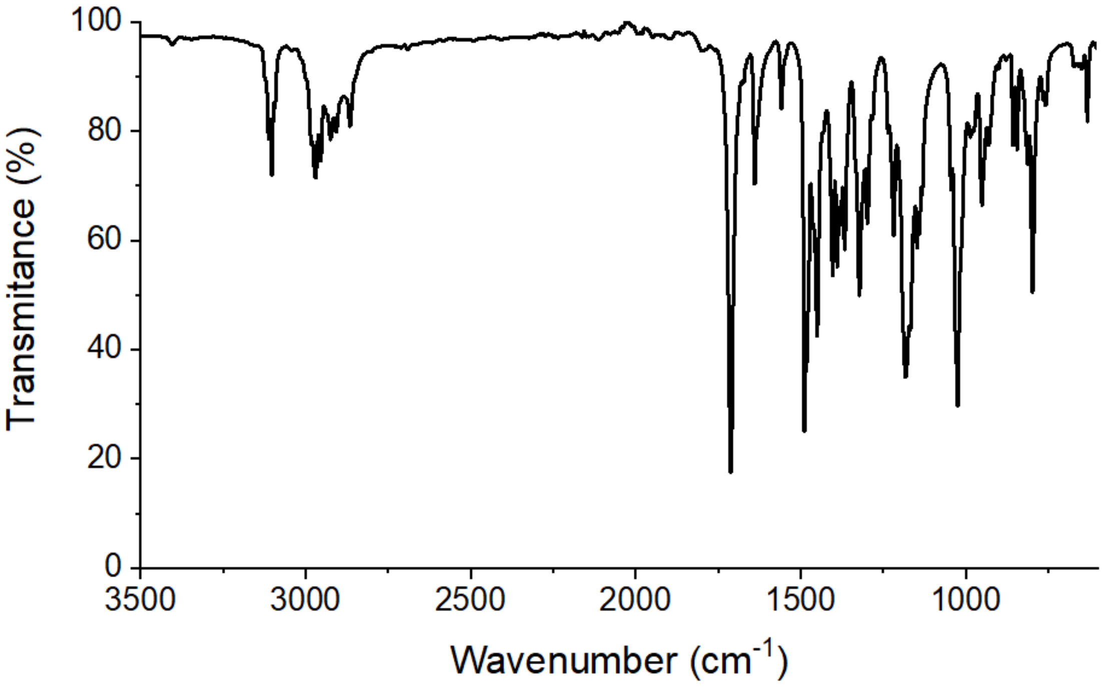

Figure S8. FT-IR spectrum of ProDOT-MM monomer.

Table S2. Assignments of major bands in FT-IR spectrum presented in *Figure S8*.

**Band position (cm^{-1})**	**Assignments and Notes**
3112	C-H stretching from 2,5 position of thiophene group
3102	
2941	C-H stretching from ProDOT ring and alkyl chains
2970	C-H stretching from ProDOT ring and alkyl chains
2955	
2925	
1712	C=O stretching from carboxyl groups

1639	C=C
1489	C=C stretching from thiophenes
1450	
1403	
1379	C-C stretching from cyclopropylene group of ProDOT
1182	C-H in plane
1044	C-O-C stretching from ProDOT ring
797	C-H out of plane

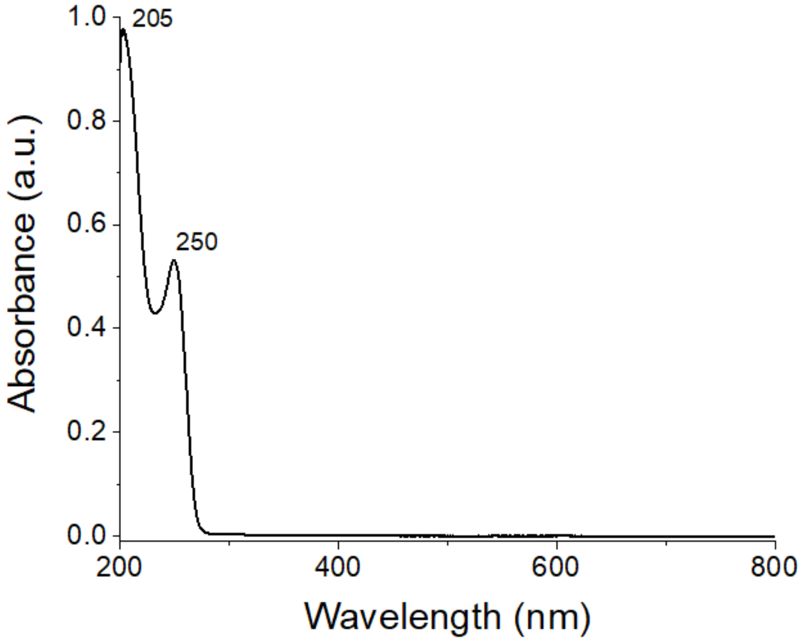

Figure S9. UV-Vis spectrum of ProDOT-MM monomer.

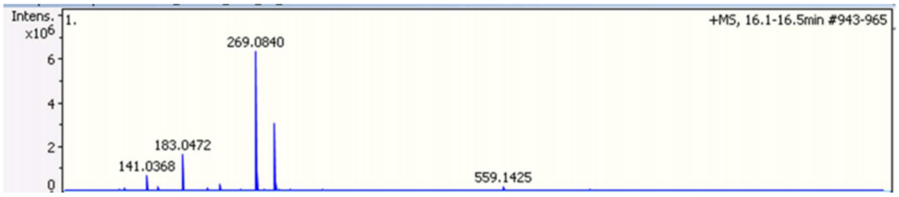

Figure S10. LCMS spectrum of ProDOT-MM monomer.

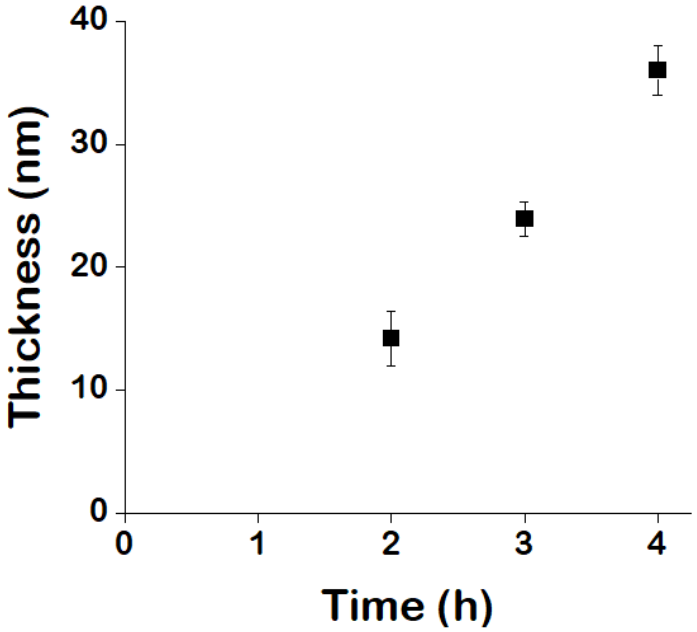

Figure S11. Dependence of the thickness of ProDOT-Poly(MM) brushes grafted from ITO on the SI-ATRP reaction time.

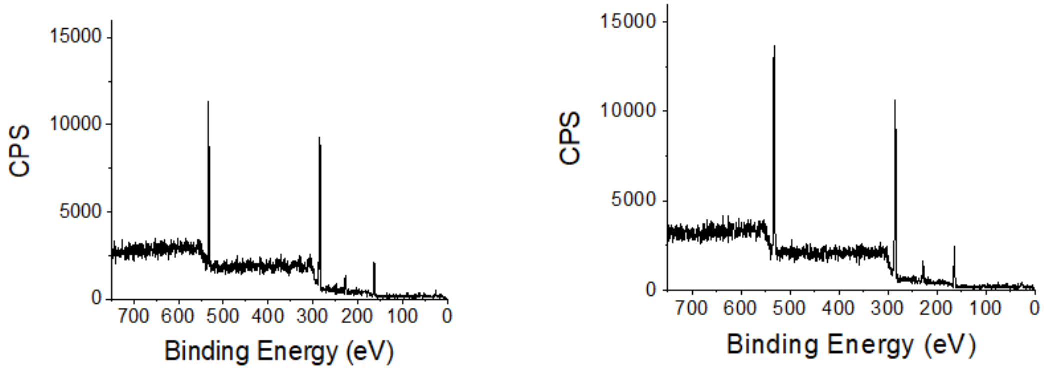

Figure S12. XPS spectra of the ProDOT-Poly(MM) (left) and Poly(ProDOT)-Poly(MM) (right) brushes grafted from quartz.

BaseLine:Constant
Chi^2=1.05277E-007 Adj. R-Square=9.99925E-001 # of Data Points=624
SS=6.41137E-005 Degrees of Freedom=609

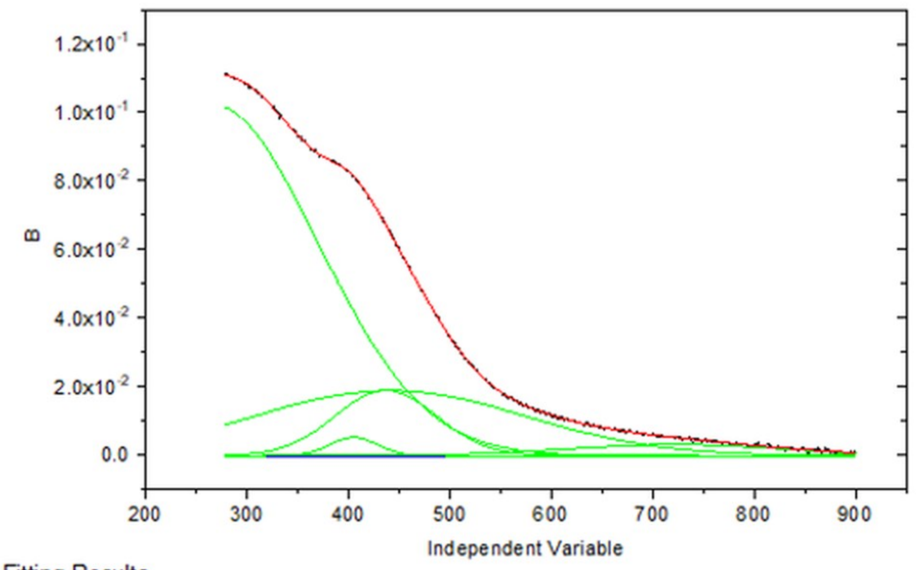

Fitting Results

Peak Index	Peak Type	Area Intg	Area IntgP	Center Grvt	Max Height	FWHM
1	Gaussian	-12.267	-57.064	264.759	0.102	247.417
2	Gaussian	-0.315	-1.466	404.764	0.005	54.927
3	Gaussian	-2.407	-11.197	436.891	0.019	118.27
4	Gaussian	-5.676	-26.405	441.014	0.019	314.989
5	Gaussian	-0.831	-3.866	722.243	0.003	253.604

Figure S13. Peak deconvolution of the absorption spectrum of the brushes after oxidative polymerization Poly(ProDOT)-Poly(MM).

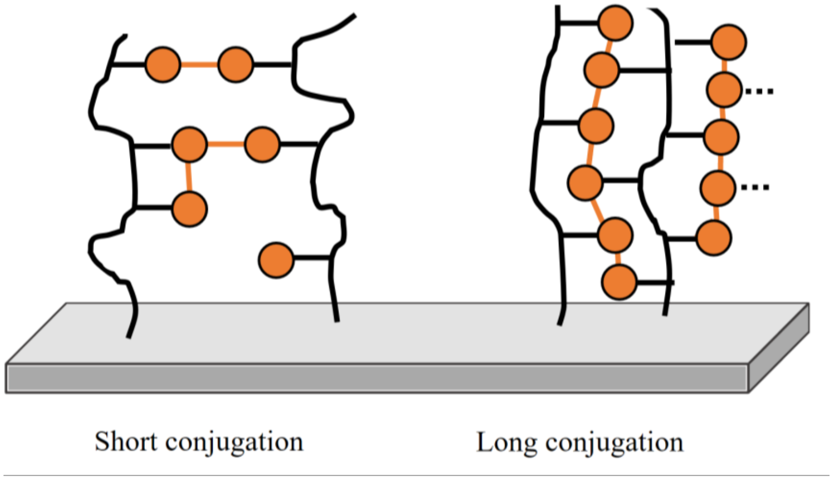

Figure S14. 2D schematic representation of conjugated chain formed in ladder-like brushes reflecting the contribution of oligomers of various lengths within single and neighbouring chains.

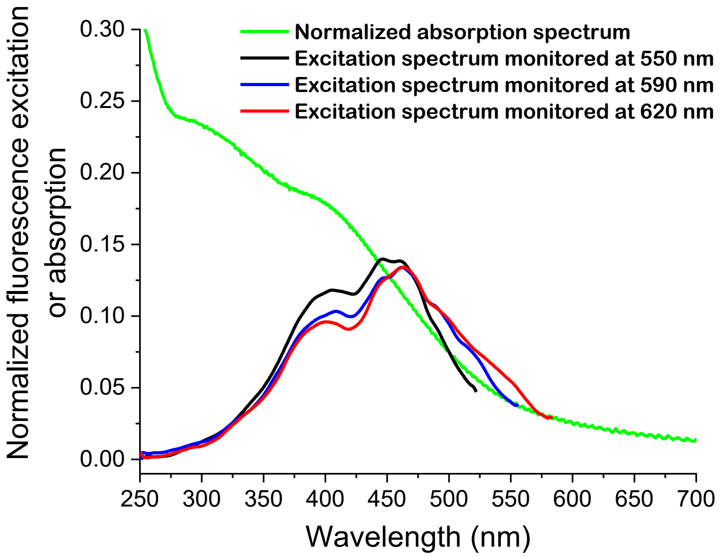

Figure S15. Fluorescence excitation spectra monitored at various emission wavelengths and absorption spectrum of Poly(ProDOT)-Poly(MM).

BaseLine:Constant

Chi^2=2.27424E-005 Adj. R-Square=9.99803E-001 # of Data Points=306

SS=6.61804E-003 Degrees of Freedom=291

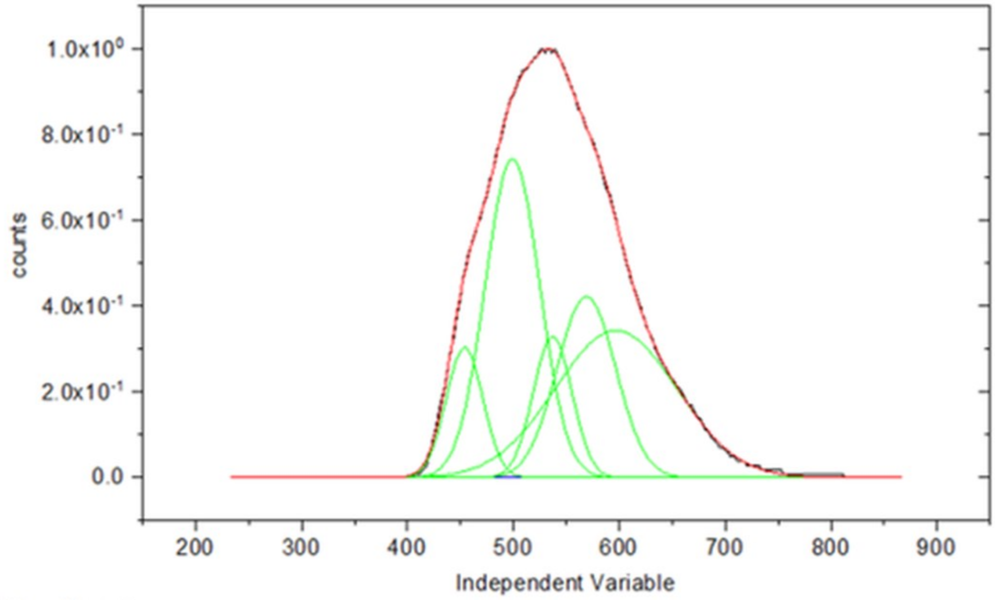

Fitting Results

Peak Inde	Peak Type	Area Intg	Area IntgP	Center Grv	Max Heigh	FWHM
1	Gaussian	13.295	8.568	453.837	0.303	41.16
2	Gaussian	47.917	30.881	499.132	0.743	60.518
3	Gaussian	15.137	9.755	536.982	0.328	43.253
4	Gaussian	29.328	18.901	568.919	0.422	65.225
5	Gaussian	49.484	31.892	596.927	0.343	135.486

Figure S16. Gaussian deconvolution of the photoluminescence spectrum of the brushes grafted from quartz after oxidative polymerization (Poly(ProDOT)-Poly(MM)).

BaseLine:Constant

Chi^2=4.18584E+003 Adj. R-Square=9.98862E-001 # of Data Points=321

SS=1.28924E+006 Degrees of Freedom=308

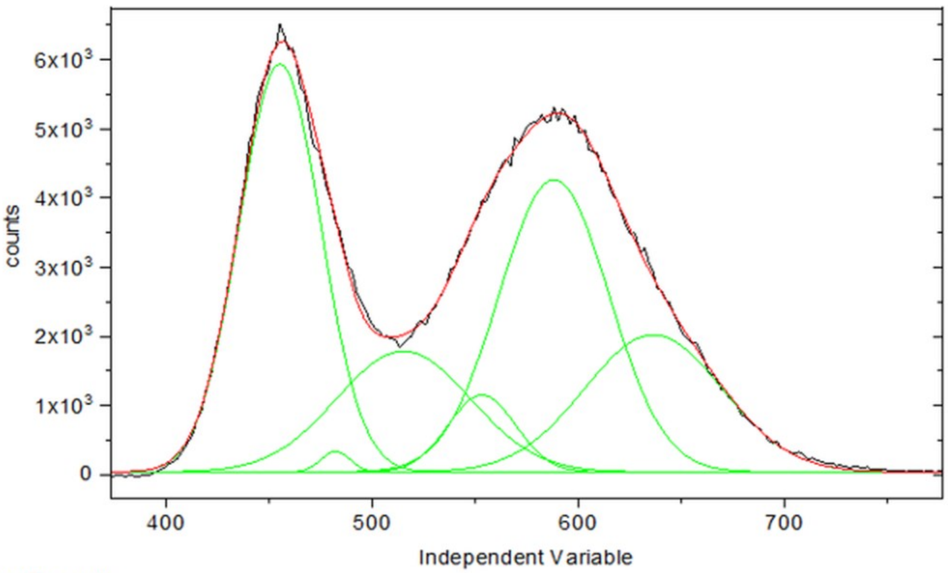

Fitting Results

Peak Index	Peak Type	Area Intg	Area IntgP	Center Grvt	Max Height	FWHM
1	Gaussian	293031.52	31.338	455	5904.961	46.619
2	Gaussian	5205.527	0.556	482	295.637	16.525
3	Gaussian	141990.97	15.185	515	1745.805	76.406
4	Gaussian	45751.918	4.892	553	1113.589	38.596
5	Gaussian	282656.98	30.229	588	4230.54	62.767
6	Gaussian	166412.31	17.797	636.2	1981.941	78.879

Figure S17. Gaussian deconvolution of photoluminescence spectra of the brushes grafted from silicon oxide (after oxidative polymerization).

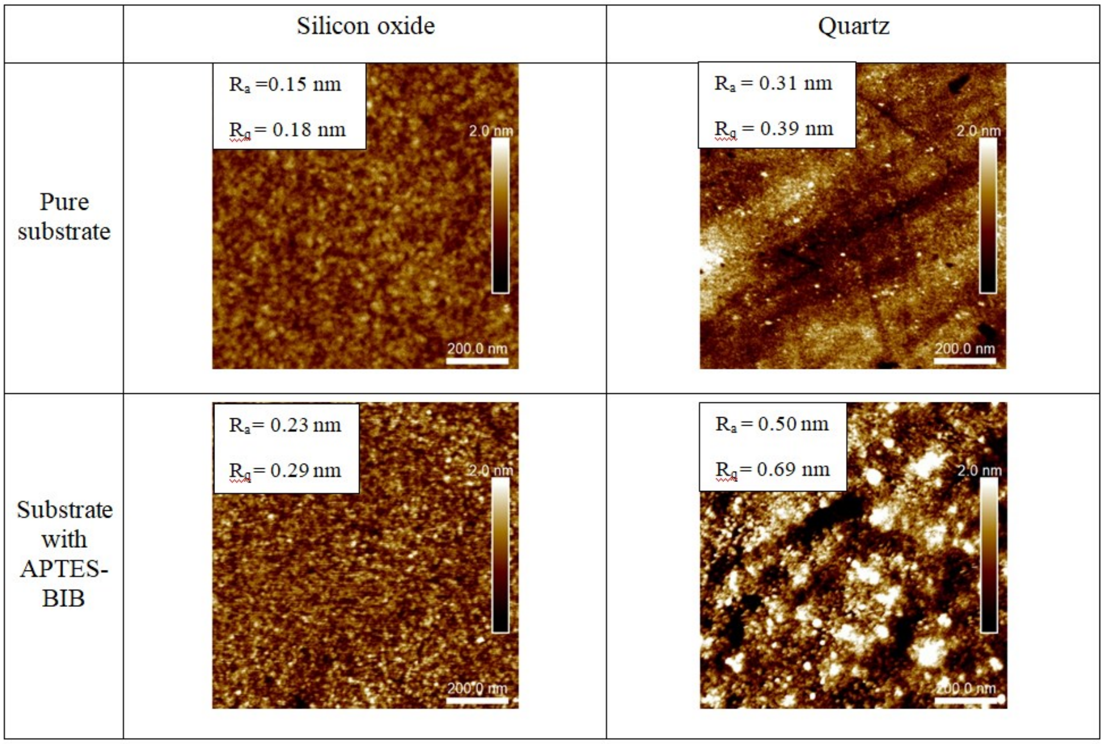

Figure S18. AFM topography images of pure silicon oxide and quartz substrates as well as coated with the APTES-BIB initiator layers. The values of mean roughness and root-mean-square roughness (R_a and R_q) are presented for each image.

Table S3. Mean fluorescence life time and its components for Poly(ProDOT)-Poly(MM) brushes grafted form quartz and silicon oxide.

	**RED BAND**							
	A_1	τ_1 [ns]	A_2	τ_2 [ns]	A_3	τ_3 [ns]	x_2	τ_I [ns]
**Quartz**	25803	**0.055**	3691	**0.39**	626	**1.72**	0.983	**0.63**
**Silicon oxide**	2448	**0.167**	/	/	/	/	1.05	**0.18**
**BLUE BAND**								
	A_1	τ_1 [ns]	A_2	τ_2 [ns]	A_3	τ_3 [ns]	x_2	τ_I [ns]
**Quartz**	4292	**0.18**	/	/	/	/	1.2	**0.18**
**Silicon oxide**	2448	**0.167**	/	/	/	/	0.983	**0.167**

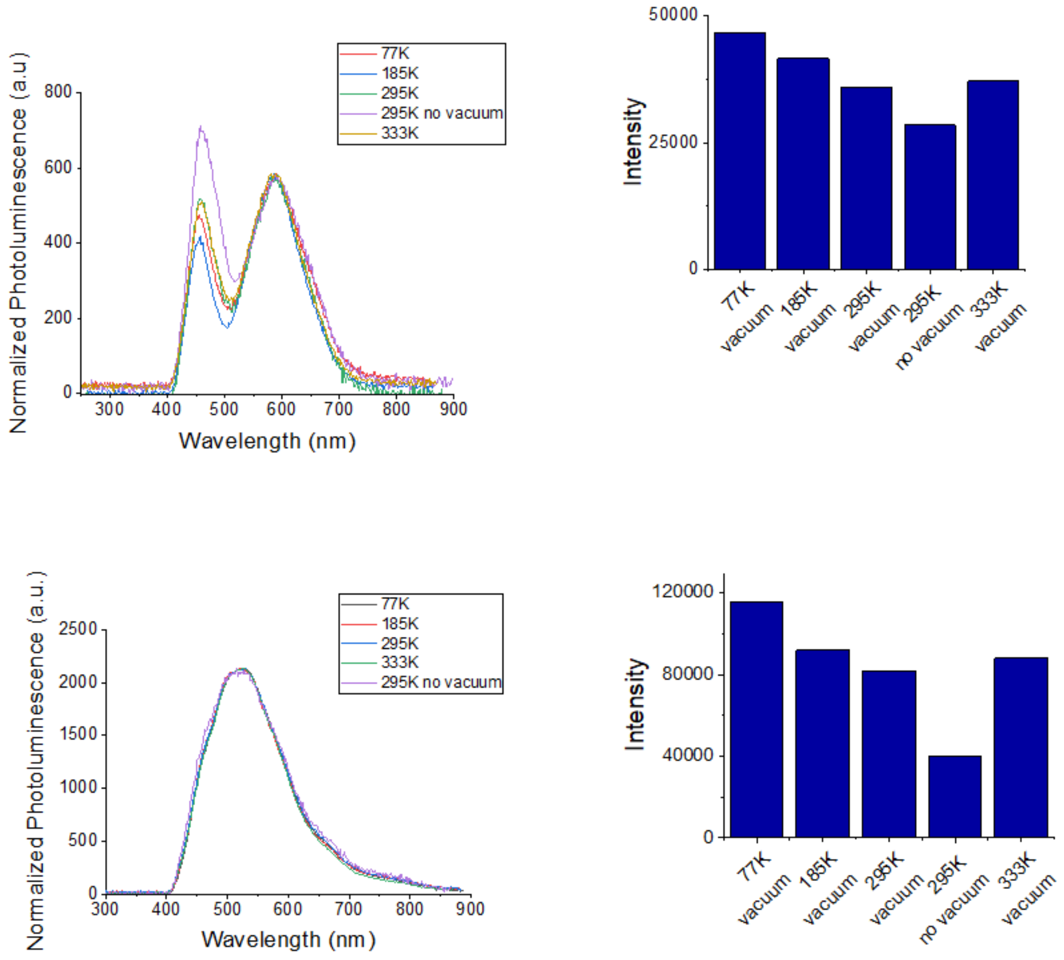

Figure S19. Normalized photoluminescence spectra of Poly(ProDOT)-Poly(MM) brushes and brightness at different temperature in vacuum and in no vacum at 295K.

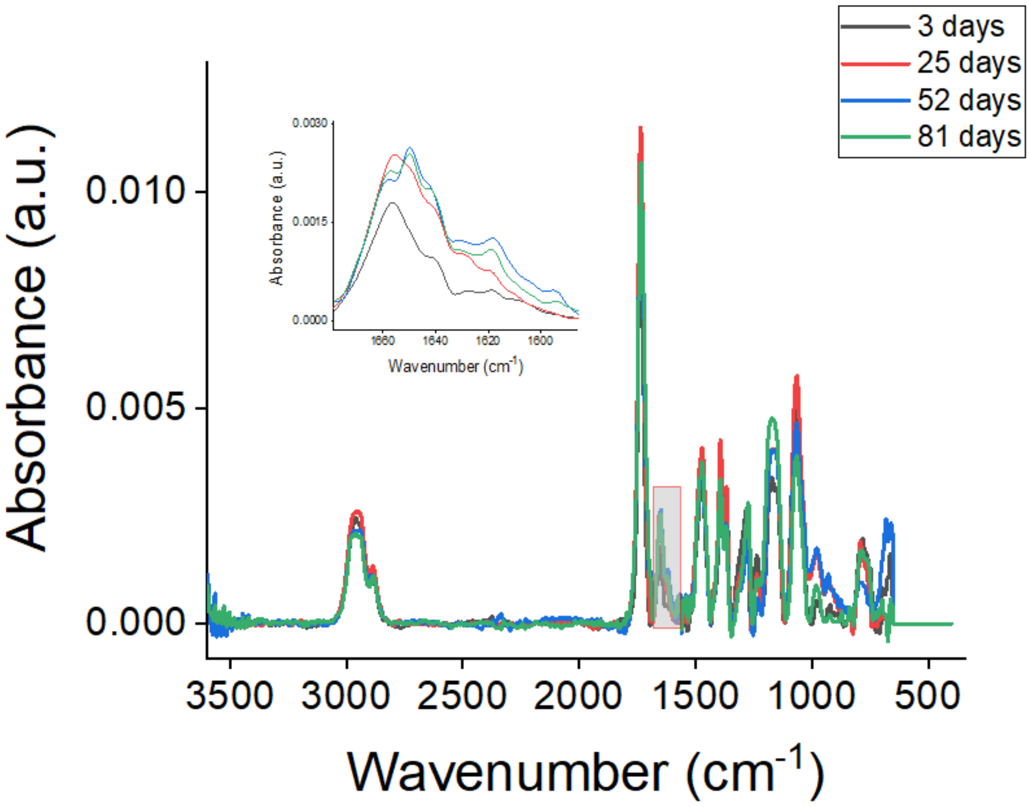

Figure S20. FT-IR spectra of the Poly(ProDOT)-Poly(MM) brushes captured after various time of storing under ambient conditions.

DFT calculations

DFT calculations enabled to verify the possibility of formation of Poly(ProDOT) chains involving one or two macromolecules in the brushes and arrangements of the ProDOT units in such a ladder-like topology. It was showed that both structures are feasible to be obtained after oxidative polymerization, although they differ in terms of the arrangements of ProDOT (see Figure S21). In the triple-stranded system (1 conjugated and 2 nonconjugated, intermolecular linking) involving two macromolecules, thiophene groups can form coplanar structures (see Figure S21), while in double-stranded ladder-like system (a single macromolecule, intramolecular linking of ProDOT groups) thiophene groups are twisted relative to the other with an average torsion angle (TA) ca.

52˚. This sterically twisted conformation is likely caused by repulsive interaction between oxygen atoms derived from adjacent ProDOT subunits.

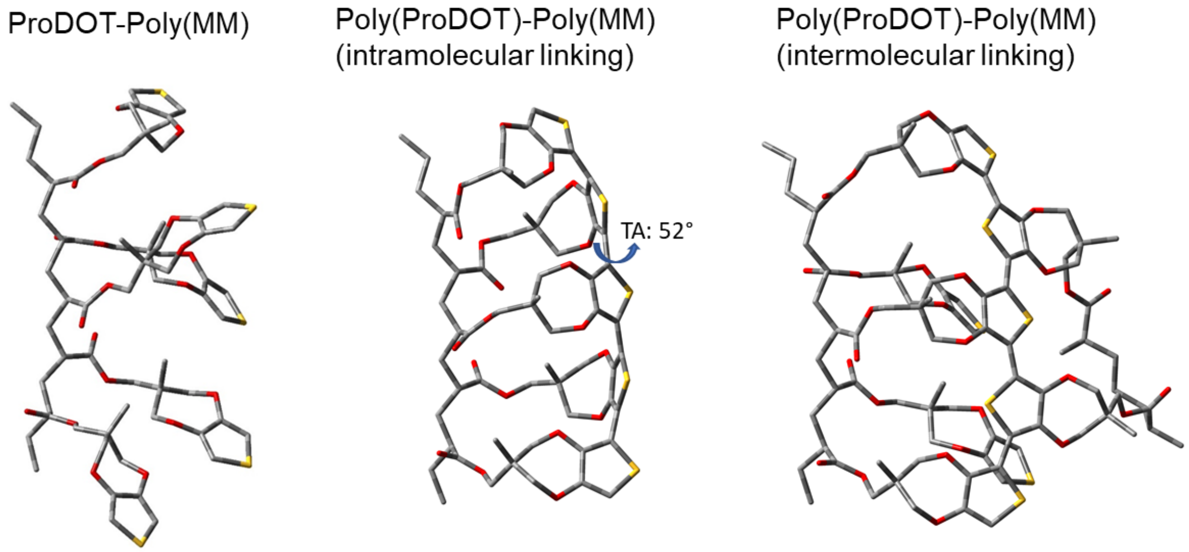

Figure S21. Molecular structures of a fragment of parent ProDOT-Poly(MM) and Poly(ProDOT)-Poly(MM) with assumed intramolecular and intermolecular linking of ProDOT groups. For clarity of the presentation the molecules were depicted without hydrogen atom.

Table S4. Optimized (B3LYP/cc-pVDZ) ground-state geometries of possible unconjugated and conjugated models from *Figure S21* along with the corresponding absolute energies. The atomic symbol followed by three Cartesian coordinates, in Å.

**ProDOT-Poly(MM)**<br>**E= -5982.610041 hartree**	**Poly(ProDOT)-Poly(MM)**<br>**(intramolecular linking)**<br>**E= -5977.742961 hartree**
C 4.750876 -2.889884 -3.106548	
C 3.259607 -3.200759 -3.350920	C -5.867679 4.123864 -1.151874
C 5.166809 -3.466743 -1.757782	C -4.418694 4.645030 -1.116773

C	2.255068	-2.477747	-2.408985	C	-6.320010	3.731514	0.253680
C	0.968313	-3.305335	-2.249633	C	-3.312677	3.572742	-0.938766
O	5.104596	-4.642169	-1.473035	C	-1.999105	4.235488	-0.474375
C	6.100492	-3.025329	0.376761	O	-6.076327	4.357850	1.261592
O	5.631004	-2.521156	-0.900461	C	-7.606222	2.077591	1.473818
C	6.468219	-1.852288	1.293358	O	-7.068397	2.600769	0.228765
C	7.158732	-2.446390	2.540119	C	-6.705344	0.969708	2.070066
C	5.215648	-1.093309	1.760142	C	-7.580861	-0.029891	2.861643
C	7.447862	-0.905813	0.559503	C	-5.647015	1.533791	3.033185
O	8.173930	-0.052013	1.463442	C	-6.035070	0.235702	0.892872
C	9.324983	-0.598102	1.971054	O	-5.504535	-1.045052	1.259696
C	10.468595	0.129525	2.169645	C	-6.401921	-2.072485	1.129285
S	11.737266	-0.847458	2.849211	C	-6.047711	-3.286011	0.579959
C	10.716428	-2.255438	2.869482	S	-7.422700	-4.377144	0.591646
C	9.468096	-1.976756	2.379974	C	-8.449048	-3.175497	1.317785
C	5.629550	-3.509065	-4.214294	C	-7.780405	-1.999156	1.537051
C	1.975293	-1.063856	-2.900129	C	-6.822561	5.210302	-1.706609
O	8.484733	-2.926612	2.276530	C	-3.058134	2.822750	-2.248033
O	0.924771	-0.647764	-3.342661	O	-8.393921	-0.874210	2.032557
C	3.017102	1.118406	-3.046617	O	-2.250967	3.160821	-3.083830
O	3.088461	-0.297165	-2.772642	C	-3.350790	0.659564	-3.259533
C	3.468380	1.892882	-1.793311	O	-3.824021	1.702010	-2.364394
C	3.488574	3.386666	-2.177571	C	-3.352895	-0.666274	-2.460494
C	-0.138544	-2.733062	-1.342507	C	-2.655350	-1.772288	-3.285252
C	4.886172	1.406908	-1.430211	C	-0.818648	3.278663	-0.195721
C	2.497260	1.647162	-0.626325	C	-4.832456	-1.086423	-2.225816
O	3.705468	4.264249	-1.067331	C	-2.566261	-0.404583	-1.158027
O	5.363658	1.892010	-0.172627	O	-2.169438	-2.889375	-2.507435
C	4.984215	4.330700	-0.571214	O	-5.051605	-2.506614	-2.168169

C	5.618920	5.524749	-0.346520	C	-2.876899	-3.341796	-1.433224
C	6.975876	3.567529	0.402614	C	-2.242706	-3.889945	-0.329369
S	7.161718	5.295378	0.421296	C	-4.739525	-3.580356	-0.008049
C	5.772713	3.200013	-0.138964	S	-3.420538	-4.262584	0.923423
C	-1.336090	-3.722320	-1.282872	C	-4.282180	-3.155429	-1.236728
C	0.386768	-2.411497	0.050678	C	0.525328	4.039850	-0.388586
O	1.438265	-2.800817	0.513511	C	-0.896040	2.708983	1.221004
O	-0.462249	-1.599085	0.736041	O	-1.491003	3.215334	2.146586
C	0.008140	-1.114425	2.017536	O	-0.173531	1.558938	1.344050
C	-1.176649	-0.732873	2.920223	C	0.008333	1.031850	2.687079
C	-0.534883	-0.060674	4.156500	C	0.254325	-0.488390	2.649507
C	-1.964123	-1.980278	3.349771	C	-1.018381	-1.166491	2.109596
C	-2.075080	0.261316	2.154027	C	0.538185	-0.957306	4.086567
O	-1.457256	0.241674	5.206676	C	1.473901	-0.775710	1.747191
O	-3.267846	0.648191	2.858060	O	-0.945805	-2.600431	2.055633
C	-3.106162	1.550963	3.879444	O	1.893579	-2.156684	1.731640
C	-3.830277	2.711498	3.968403	C	1.173626	-2.950976	0.875220
S	-3.490153	3.561353	5.446734	C	1.704156	-3.570116	-0.245395
C	-2.328487	2.362814	5.932279	S	0.426646	-4.367096	-1.151949
C	-2.237497	1.350303	5.013618	C	-0.815701	-3.818986	-0.041774
C	-2.731063	-3.100338	-1.039227	C	-0.236917	-3.121732	1.007060
C	-3.794871	-4.208651	-0.945148	C	1.842554	3.216905	-0.398004
C	-3.050219	-2.159939	-2.194941	C	3.056478	4.159725	-0.222860
O	-3.376714	-2.512717	-3.306327	C	1.871578	2.290923	-1.612170
O	-2.888087	-0.858090	-1.848461	O	0.972982	1.525603	-1.904954
C	-3.057044	0.106100	-2.913207	O	3.021313	2.346126	-2.324669
C	-2.879640	1.518924	-2.333454	C	3.221671	1.307818	-3.318957
C	-3.038228	2.486593	-3.525409	C	3.564320	-0.092665	-2.700730
C	-1.456674	1.611390	-1.743805	C	4.635367	-0.748852	-3.613766

C	-3.945353	1.810572	-1.265537	C	2.293972	-1.002812	-2.703063
O	-3.076747	3.866191	-3.152809	C	4.137003	0.138657	-1.287100
O	-1.175881	2.828964	-1.036681	O	5.375200	-1.861977	-3.059044
C	-1.886149	4.446295	-2.806426	O	2.585033	-2.399567	-2.909272
C	-1.478576	5.640711	-3.341978	C	4.837820	-2.606285	-2.051424
C	0.078502	4.802882	-1.581776	C	5.553067	-2.930687	-0.904953
S	-0.016008	6.210986	-2.598563	C	3.062390	-3.443905	-0.753479
C	-0.982487	3.957480	-1.790123	S	4.476108	-3.707696	0.255140
C	7.116924	-3.170778	-4.090447	C	3.445701	-2.891011	-1.958925
C	-5.207025	-3.725966	-0.575941	C	-8.281630	4.773981	-1.855985
C	-6.227610	-4.883522	-0.702048	C	4.436625	3.571382	0.156832
C	-5.222000	-3.173950	0.841895	C	5.534435	4.673903	0.123985
O	-4.623647	-3.653926	1.783112	C	4.410504	2.854487	1.505348
C	-7.672195	-4.537731	-0.321696	O	3.413033	2.636268	2.165602
O	-6.011281	-2.082735	0.942433	C	6.923626	4.195903	-0.318307
C	-6.165081	-1.514897	2.263711	O	5.656206	2.471224	1.896899
C	-7.219883	-0.403814	2.210064	C	5.802351	1.853556	3.200475
C	-7.441519	0.105381	3.645604	C	6.312915	0.395058	3.150562
C	-8.578100	-0.916064	1.666795	C	6.140619	-0.191121	4.567602
C	-6.690593	0.731511	1.314393	C	7.822725	0.265785	2.821648
O	-8.672598	-0.946848	0.236145	C	5.464704	-0.401205	2.138329
O	-7.670014	1.760093	1.100024	O	8.199782	0.489443	1.460818
C	-9.024881	0.242432	-0.347242	O	6.095243	-1.622798	1.735279
C	-8.649020	-5.667330	-0.657114	C	8.000204	-0.542107	0.579007
C	-9.015916	2.554493	-0.724156	C	7.959325	5.322286	-0.319069
C	-9.879260	0.315606	-1.415753	C	6.784296	-2.264393	-0.491667
S	-10.092785	1.955981	-1.952622	C	8.737698	-0.672012	-0.570403
C	-8.522180	1.538385	0.051230	S	8.084798	-1.899559	-1.614923
H	4.906884	-1.803478	-3.085669	C	6.936506	-1.502516	0.659377

H	3.121437	-4.285882	-3.219238	H	-5.942476	3.230736	-1.791324
H	3.011724	-2.977426	-4.402417	H	-4.338625	5.357985	-0.281516
H	2.728201	-2.377097	-1.420702	H	-4.214236	5.208211	-2.042544
H	1.266948	-4.293992	-1.865280	H	-3.646252	2.842071	-0.184301
H	0.520722	-3.465813	-3.244201	H	-2.209719	4.812400	0.438205
H	6.975935	-3.669458	0.198287	H	-1.695127	4.944018	-1.259396
H	5.308983	-3.644953	0.825739	H	-8.574197	1.648467	1.180399
H	6.594862	-3.319861	2.903032	H	-7.754088	2.904055	2.183269
H	7.186471	-1.699133	3.349482	H	-8.289276	0.515450	3.504932
H	5.499409	-0.178633	2.302181	H	-6.942103	-0.650687	3.510516
H	4.598243	-1.720349	2.424936	H	-4.946280	0.738892	3.334394
H	4.591783	-0.799620	0.904334	H	-6.117168	1.939207	3.944691
H	8.163255	-1.478788	-0.053352	H	-5.074074	2.350747	2.572425
H	6.894381	-0.221904	-0.093537	H	-6.742542	0.112772	0.059086
H	10.627619	1.179467	1.939197	H	-5.180664	0.818172	0.524393
H	11.073102	-3.209587	3.247727	H	-9.490478	-3.387200	1.545345
H	5.249552	-3.152921	-5.187445	H	-6.430949	5.519251	-2.690946
H	5.491191	-4.603482	-4.199731	H	-6.758119	6.097664	-1.052608
H	1.991451	1.381273	-3.338369	H	-2.341414	0.929443	-3.600700
H	3.690925	1.309066	-3.897839	H	-4.022421	0.622622	-4.131298
H	2.507814	3.685620	-2.575532	H	-1.742470	-1.376126	-3.754110
H	4.252831	3.576186	-2.954678	H	-3.320923	-2.147894	-4.080389
H	-0.489785	-1.785243	-1.772640	H	-0.814434	2.438084	-0.900243
H	5.600770	1.678832	-2.230699	H	-5.449081	-0.766887	-3.078941
H	4.883650	0.315153	-1.323853	H	-5.226696	-0.612055	-1.318994
H	2.830358	2.185294	0.270274	H	-2.551982	-1.274576	-0.498028
H	2.453762	0.575162	-0.378352	H	-3.022476	0.419542	-0.596699
H	1.481070	1.991255	-0.873601	H	-1.524613	-0.127650	-1.389670
H	5.238329	6.518593	-0.566792	H	0.609591	4.799625	0.406478

H	7.727257	2.898138	0.815090	H	0.449137	4.593841	-1.339072
H	-1.151199	-4.487759	-0.510847	H	-0.881775	1.272643	3.285400
H	-1.375722	-4.256649	-2.246448	H	0.875287	1.550773	3.127379
H	0.653750	-0.241198	1.818641	H	-1.864960	-0.955680	2.780470
H	0.624318	-1.891832	2.489788	H	-1.259103	-0.772580	1.111974
H	0.188972	-0.751977	4.614701	H	0.689206	-2.044648	4.104635
H	0.003783	0.859351	3.861857	H	-0.304461	-0.715472	4.754509
H	-2.758468	-1.710791	4.057952	H	1.443817	-0.475922	4.490508
H	-1.297849	-2.701049	3.851821	H	2.341099	-0.217000	2.129332
H	-2.433807	-2.486232	2.495016	H	1.275178	-0.440218	0.716457
H	-2.441006	-0.212892	1.234907	H	1.812044	2.534158	0.463302
H	-1.507001	1.167510	1.872672	H	2.782756	4.890493	0.556113
H	-4.550185	3.098668	3.251969	H	3.196199	4.735411	-1.151703
H	-1.792775	2.442168	6.874497	H	4.075376	1.687987	-3.895628
H	-2.702837	-2.514199	-0.110547	H	2.337790	1.259655	-3.969517
H	-3.470981	-4.938693	-0.187070	H	4.177248	-1.073998	-4.563181
H	-3.844276	-4.726623	-1.915804	H	5.419735	-0.011048	-3.842520
H	-4.059110	-0.017746	-3.352587	H	1.641865	-0.739742	-3.546580
H	-2.314384	-0.110935	-3.698486	H	1.717176	-0.879215	-1.774832
H	-2.234264	2.322381	-4.267413	H	4.413250	-0.794305	-0.795484
H	-4.005286	2.310160	-4.020383	H	3.400488	0.627539	-0.637940
H	-0.705513	1.454977	-2.539346	H	5.036363	0.772285	-1.331432
H	-1.322674	0.823936	-0.991634	H	-8.880648	5.578507	-2.311255
H	-3.819205	2.820929	-0.855544	H	-8.741601	4.526716	-0.886262
H	-3.876992	1.089480	-0.438607	H	-8.370202	3.882968	-2.499453
H	-4.957285	1.742573	-1.697204	H	4.714094	2.810940	-0.588931
H	-1.990174	6.219175	-4.106667	H	5.603398	5.162566	1.112538
H	0.888356	4.689630	-0.865127	H	5.197650	5.459966	-0.574006
H	7.690425	-3.610287	-4.921715	H	6.842486	3.770379	-1.336058

H	7.547124	-3.561199	-3.153404	H	7.261335	3.375326	0.332769
H	7.285465	-2.080773	-4.106543	H	4.826142	1.891818	3.701242
H	-5.526452	-2.917319	-1.252478	H	6.515552	2.483601	3.754011
H	-5.876290	-5.732012	-0.087204	H	6.380670	-1.265218	4.564308
H	-6.199840	-5.227437	-1.751210	H	5.104050	-0.076631	4.921013
H	-7.981942	-3.607169	-0.827508	H	6.803011	0.304533	5.297156
H	-7.727888	-4.320380	0.759711	H	8.172263	-0.731736	3.135095
H	-5.194604	-1.114613	2.597408	H	8.383472	1.017619	3.397844
H	-6.461959	-2.311904	2.963819	H	5.262311	0.205463	1.246183
H	-8.061509	1.014231	3.631210	H	4.505084	-0.698342	2.585757
H	-6.484748	0.352321	4.132338	H	8.942670	4.961371	-0.660299
H	-7.951540	-0.650672	4.265688	H	8.093238	5.743789	0.692027
H	-9.397524	-0.295187	2.066642	H	7.658477	6.150407	-0.983913
H	-8.748364	-1.952787	1.994353	H	9.609672	-0.091586	-0.859374
H	-6.350847	0.331878	0.347390				
H	-5.836465	1.220704	1.804302				
H	-9.678027	-5.409604	-0.359670				
H	-8.376074	-6.602978	-0.139546				
H	-8.658435	-5.880333	-1.739761				
H	-8.789804	3.613620	-0.635478				
H	-10.393703	-0.510524	-1.899133				
**Poly(ProDOT)-Poly(MM)**							
**(intermolecular linking)**							
**E= -8426.644356 hartree**							
C	0.999679	7.180078	2.305134				
C	-0.506953	6.937467	2.098413				
C	1.677625	7.587313	1.001180				

C	-0.938268	5.605815	1.419880	
C	-2.437380	5.686682	1.080630	
O	1.272923	8.454482	0.258280	
C	3.748033	7.366231	-0.223735	
O	2.836719	6.907336	0.811473	
C	3.660426	6.561254	-1.534508	
C	2.528607	7.049040	-2.453833	
C	3.474630	5.066396	-1.200349	
O	3.864836	4.235150	-2.306084	
C	5.182276	3.857758	-2.270955	
C	5.570939	2.546629	-2.481517	
S	7.325420	2.424349	-2.356166	
C	7.482004	4.127192	-2.008624	
C	6.264142	4.755408	-1.988498	
C	1.219963	8.316091	3.336011	
C	-0.660366	4.433641	2.352432	
C	4.998827	6.750407	-2.289418	
O	6.112371	6.083202	-1.674453	
O	-1.481539	3.888782	3.061164	
C	1.101965	3.041416	3.219361	
O	0.649819	4.079654	2.319043	
C	2.121462	2.142396	2.501559	
C	2.589384	1.114691	3.552617	
C	-3.074928	4.495316	0.341578	
C	3.305759	3.020717	2.051868	
C	1.468796	1.435694	1.301779	
O	3.381189	0.054052	3.010003	
O	4.242943	2.349032	1.206772	
C	4.673591	0.358219	2.665215	

C	5.726899	-0.431477	3.048566	
C	6.429956	1.440685	1.556436	
S	7.221124	0.107333	2.342150	
C	5.081818	1.446740	1.804209	
C	-4.626570	4.646438	0.296772	
C	-2.525056	4.358074	-1.073644	
O	-1.679634	5.068193	-1.574002	
O	-3.138196	3.345102	-1.741813	
C	-2.970467	3.278984	-3.182685	
C	-2.169811	2.056312	-3.664659	
C	-0.685073	2.220798	-3.279053	
C	-2.313529	2.006185	-5.196951	
C	-2.768881	0.790664	-3.021170	
O	0.178081	1.256314	-3.899383	
O	-2.254151	-0.431074	-3.565479	
C	-0.991299	-0.803134	-3.182091	
S	0.997470	-2.273520	-2.419100	
C	0.175578	-0.009433	-3.374023	
C	-5.446019	3.347629	0.481231	
C	-6.938892	3.626873	0.221578	
C	-5.284137	2.865872	1.919097	
O	-5.807304	3.394459	2.876437	
O	-4.486102	1.775964	2.021988	
C	-4.300270	1.278711	3.369695	
C	-3.554260	-0.063947	3.324650	
C	-3.530771	-0.566150	4.785648	
C	-2.124777	0.212618	2.817351	
C	-4.277581	-1.077920	2.425037	
O	-2.991860	-1.879694	4.948302	

O	-1.310556	-0.950802	2.630728	
C	-1.629820	-1.994383	4.873940	
C	-0.895480	-2.639555	5.834788	
C	0.501282	-1.876531	3.909922	
S	0.781138	-2.747122	5.390398	
C	-0.823320	-1.551513	3.760941	
C	2.686090	8.614253	3.657246	
C	-7.871404	2.397870	0.341879	
C	-9.190905	2.788652	1.060415	
C	-8.196660	1.824909	-1.031676	
O	-8.336322	2.477607	-2.042529	
C	-10.197495	1.658658	1.299744	
O	-8.369137	0.474997	-0.988225	
C	-8.845941	-0.150085	-2.206060	
C	-8.387622	-1.613105	-2.285318	
C	-8.896541	-2.181049	-3.622204	
C	-8.987377	-2.409863	-1.105733	
C	-6.847603	-1.634410	-2.241428	
O	-8.794917	-3.828455	-1.197154	
O	-6.283563	-2.922223	-2.514654	
C	-7.545484	-4.294908	-0.880073	
C	-11.401098	2.121357	2.125176	
C	-7.325431	-5.300597	0.024510	
C	-6.335552	-3.845955	-1.504015	
C	4.702376	1.413136	-2.691207	
S	2.969732	1.626822	-2.949817	
C	2.686047	-0.110816	-2.986503	
C	3.885806	-0.789013	-2.856736	
C	1.350512	-0.654217	-3.017461	

O	6.300885	-0.385480	-2.493024	
C	5.120320	-2.705401	-3.486340	
O	3.944925	-2.159301	-2.867499	
C	5.017619	0.064171	-2.677022	
C	6.280856	-2.861152	-2.482482	
C	5.902345	-4.033394	-1.567867	
C	6.415175	-1.558379	-1.659624	
C	7.575338	-3.146093	-3.257980	
O	6.803053	-4.133730	-0.441036	
C	7.216091	-5.381878	-0.087149	
C	7.247902	-6.045605	2.346811	
C	8.026508	-5.358229	1.198514	
O	6.951314	-6.383415	-0.714793	
C	9.378854	-6.047002	0.961221	
C	5.883826	-5.416896	2.704237	
C	4.678041	-6.069990	2.009049	
C	3.339094	-5.358619	2.294882	
C	2.166858	-6.302246	2.008941	
C	3.210555	-3.993850	1.607132	
C	-3.827777	-4.197695	-1.416687	
C	-1.474008	-4.145107	-1.399999	
S	-5.638673	-5.721501	0.129285	
S	-3.436109	-2.793319	-2.413726	
O	2.151745	-7.484356	2.279004	
C	-1.704116	-3.020061	-2.176083	
O	-2.693384	-5.839906	-0.071150	
C	-0.738970	-2.047460	-2.621375	
C	-2.664674	-4.803495	-0.969091	
O	-0.213556	-4.479087	-0.993175	

C	-1.899112	-7.001392	-0.358110	
C	-0.377903	-6.823790	-0.155283	
C	0.195154	-5.837125	-1.187140	
C	-0.138810	-6.325052	1.288474	
C	0.285136	-8.192252	-0.402693	
O	1.124162	-5.645566	1.451454	
C	-5.194762	-4.491622	-1.054430	
H	1.493024	6.271431	2.673905	
H	-0.897786	7.764176	1.484017	
H	-1.012600	7.003342	3.076959	
H	-0.349313	5.465395	0.501517	
H	-2.597845	6.596877	0.481453	
H	-2.987508	5.815020	2.025656	
H	4.742595	7.222942	0.221532	
H	3.566470	8.434636	-0.409448	
H	2.444301	6.387389	-3.330459	
H	2.719244	8.074521	-2.811160	
H	1.562479	7.060686	-1.930366	
H	4.053502	4.772859	-0.312218	
H	2.415385	4.843011	-1.012290	
H	8.456493	4.577559	-1.840342	
H	0.693031	8.028153	4.261975	
H	0.723918	9.228266	2.961677	
H	4.890892	6.413142	-3.332274	
H	5.273697	7.816695	-2.303362	
H	0.233733	2.463729	3.562110	
H	1.554086	3.542716	4.092544	
H	1.720503	0.606871	3.995459	
H	3.149662	1.616415	4.364218	

H	-2.835685	3.563283	0.879738	
H	3.829196	3.434925	2.935360	
H	2.937074	3.859552	1.449443	
H	2.205787	0.840745	0.748479	
H	1.044751	2.173910	0.604512	
H	0.664285	0.759276	1.631491	
H	5.685424	-1.308083	3.689591	
H	6.975668	2.123115	0.909866	
H	-4.931497	5.114927	-0.652803	
H	-4.929881	5.343332	1.094427	
H	-2.497895	4.209264	-3.525856	
H	-3.995588	3.220594	-3.578305	
H	-0.312989	3.192843	-3.632286	
H	-0.557096	2.180510	-2.182907	
H	-1.742799	1.166273	-5.611327	
H	-1.936470	2.934539	-5.656257	
H	-3.368862	1.882193	-5.489402	
H	-3.850123	0.747873	-3.223286	
H	-2.620855	0.804340	-1.928439	
H	-5.064039	2.575246	-0.201431	
H	-7.059923	4.061070	-0.781964	
H	-7.260217	4.388976	0.947578	
H	-5.289916	1.163724	3.838043	
H	-3.742880	2.038025	3.941760	
H	-2.980790	0.144928	5.431031	
H	-4.561675	-0.645088	5.162782	
H	-1.618323	0.920454	3.499211	
H	-2.177110	0.674276	1.822335	
H	-3.755578	-2.043337	2.431666	

H	-4.324319	-0.717730	1.387316	
H	-5.308513	-1.245286	2.778624	
H	-1.268439	-3.078417	6.756391	
H	1.317133	-1.680638	3.218240	
H	2.762139	9.380144	4.445181	
H	3.232300	8.993022	2.778424	
H	3.212906	7.712873	4.012219	
H	-7.395551	1.594996	0.928341	
H	-9.671234	3.601807	0.486094	
H	-8.899278	3.221914	2.032692	
H	-9.692602	0.821867	1.815381	
H	-10.550265	1.251293	0.336833	
H	-8.473725	0.424018	-3.066028	
H	-9.947424	-0.088735	-2.209649	
H	-8.604650	-3.233028	-3.729538	
H	-8.478821	-1.619571	-4.473362	
H	-9.995878	-2.123855	-3.680173	
H	-10.080882	-2.282370	-1.099610	
H	-8.589692	-2.037651	-0.145764	
H	-6.481656	-1.264890	-1.269054	
H	-6.447405	-0.984615	-3.034881	
H	-12.115875	1.299229	2.289045	
H	-11.942712	2.938987	1.619759	
H	-11.088351	2.495143	3.114876	
H	-8.072155	-5.827792	0.612335	
H	4.819927	-3.688651	-3.879772	
H	5.421949	-2.072689	-4.335664	
H	5.920827	-4.982887	-2.120789	
H	4.892842	-3.873069	-1.158428	

H	7.407411	-1.496706	-1.193864	
H	5.660594	-1.509299	-0.859144	
H	8.389888	-3.413691	-2.567303	
H	7.886304	-2.255992	-3.826898	
H	7.445183	-3.981480	-3.965727	
H	7.907210	-6.013401	3.230250	
H	7.116770	-7.112853	2.096641	
H	8.188547	-4.302611	1.468406	
H	9.967903	-6.067403	1.891559	
H	9.970751	-5.520448	0.195602	
H	9.226495	-7.082264	0.619765	
H	5.728800	-5.496672	3.794793	
H	5.917896	-4.335444	2.484387	
H	4.593233	-7.114715	2.347004	
H	4.833494	-6.113300	0.917351	
H	3.274771	-5.203919	3.391078	
H	2.269805	-3.496604	1.875673	
H	3.216940	-4.099916	0.510310	
H	4.043146	-3.332483	1.889760	
H	-2.280620	-7.764402	0.336700	
H	-2.099253	-7.343133	-1.389801	
H	-0.071295	-6.165610	-2.208378	
H	1.289150	-5.810467	-1.104695	
H	-0.193204	-7.164177	1.996912	
H	-0.887923	-5.567166	1.545173	
H	1.365302	-8.151858	-0.204169	
H	0.134680	-8.526086	-1.443013	
H	-0.138064	-8.958930	0.264896	
